# Genome sequence and transcriptome analyses of the thermophilic zygomycete fungus *Rhizomucor miehei*

**DOI:** 10.1186/1471-2164-15-294

**Published:** 2014-04-21

**Authors:** Peng Zhou, Guoqiang Zhang, Shangwu Chen, Zhengqiang Jiang, Yanbin Tang, Bernard Henrissat, Qiaojuan Yan, Shaoqing Yang, Chin-Fu Chen, Bing Zhang, Zhenglin Du

**Affiliations:** 1 Department of Biotechnology, College of Food Science and Nutritional Engineering, China Agricultural University, Beijing 100083, China; 2 Core Genomic Facility, Beijing Institute of Genomics, Chinese Academy of Sciences, Beijing 100101, China; 3 UMR 7257 - Centre National de la Recherche Scientifique & Aix-Marseille Université, Marseille 13288, France; 4 Department of Biological Sciences, King Abdulazziz University, Jeddah, Saudi Arabia; 5 Bioresource Utilization Laboratory, College of Engineering, China Agricultural University, Beijing 100083, China; 6 Office of Bioinformatics and Center for Molecular Studies, 113 Gregor Mendel Circle Greenwood, SC 29646, USA

**Keywords:** *Rhizomucor miehei*, Genome, Transcriptome, Thermophilic fungus, Thermostable enzymes

## Abstract

**Background:**

The zygomycete fungi like *Rhizomucor miehei* have been extensively exploited for the production of various enzymes. As a thermophilic fungus, *R. miehei* is capable of growing at temperatures that approach the upper limits for all eukaryotes. To date, over hundreds of fungal genomes are publicly available. However, Zygomycetes have been rarely investigated both genetically and genomically.

**Results:**

Here, we report the genome of *R. miehei* CAU432 to explore the thermostable enzymatic repertoire of this fungus. The assembled genome size is 27.6-million-base (Mb) with 10,345 predicted protein-coding genes. Even being thermophilic, the G + C contents of fungal whole genome (43.8%) and coding genes (47.4%) are less than 50%. Phylogenetically, *R. miehei* is more closerly related to *Phycomyces blakesleeanus* than to *Mucor circinelloides* and *Rhizopus oryzae*. The genome of *R. miehei* harbors a large number of genes encoding secreted proteases, which is consistent with the characteristics of *R. miehei* being a rich producer of proteases. The transcriptome profile of *R. miehei* showed that the genes responsible for degrading starch, glucan, protein and lipid were highly expressed.

**Conclusions:**

The genome information of *R. miehei* will facilitate future studies to better understand the mechanisms of fungal thermophilic adaptation and the exploring of the potential of *R. miehei* in industrial-scale production of thermostable enzymes. Based on the existence of a large repertoire of amylolytic, proteolytic and lipolytic genes in the genome, *R. miehei* has potential in the production of a variety of such enzymes.

## Background

Of the predicted about 0.6 million fungal species, only 44,368 have been catalogued [1]. Seven major groups (“Phyla”), 10 subphyla, 35 classes, 12 subclasses and 129 orders are recognized within the fungal kingdom [2]. Thermophilic fungi in general have a maximum growth temperature at or above 40°C [3]. This is in contrast to most other mesophilic fungi by displaying a maximum temperature less than 35°C. Among more than forty thermophilic fungi which have been studied [3], five (viz. *Rhizomucor miehei*, *Rhizomucor nainitalensis*, *Rhizomucor pusillus*, *Rhizopus microsporus*, *Rhizopus rhizopodiformis*) belong to Zygomycetes. The genera *Rhizomucor*, *Mucor* and *Rhizopus* are classified under the family Mucoraceae in the order Mucorales, which is a primitive and early divergent group of fungi. The genus *Rhizomucor* consists of Mucor-like fungi that produce nonapophysate sporangia and branched sporangiophores but unlike *Mucor* they form rhizoids. *Rhizomucor* species are clearly distinct from *Mucor* by virtue of their thermophilic nature and some morphological features [4]. The genus *Rhizomucor*, as monographed by Schipper [4], contains two well-known species (*R. miehei* and *R. pusillus*) that are thermophilic in nature and can grow at temperatures of 50°C or above [5].

Thermophilic fungi are important producers of thermostable enzymes that can be used in industrial high-temperature bioprocesses [3,6]. For a long time, *R. miehei* as a thermophilic fungus has been used mainly as a producer of industrial proteases and lipases [6-9]. The aspartic protease produced by *R. miehei* has been widely used as a calf chymosin substitute in industrial cheese making [7,9]. The lipases from *R. miehei* are well studied and commercially available in both soluble and immobilized forms with very high activities and good stabilities [6,8]. Other extracellular enzymes from *R. miehei* have been characterized but have not been used commercially including fibrinolytic enzyme, β-glucanase and xylanase [10-12]. Despite their potential industrial values, most members of the genus *Rhizomucor* remain poorly characterized [13].

Currently, a few hundreds of fungal genomes have been sequenced, including important human pathogens, plant pathogens and model organisms [14-20]. The genome of Industry-related fungi, like *Aspergillus niger* (which is widely used for the production of enzymes) and *Trichoderma reesei* (an industrial producer of plant biomass hydrolyzing enzymes), have also been sequenced [21,22]. Comparative genomic analyses of three thermophilic ascomycete species, *Thermomyces lanuginosus *[20], *Thielavia terrestris* and *Myceliophthora thermophila* suggest that aside from representing a potential reservoir of thermostable enzymes, thermophilic fungi are amenable to be manipulated using classical and molecular genetics [23]. A thermophilic fungus *R. miehei* strain CAU432, newly isolated from self-heating hay in Henan Province of China, has been found to be a good producer of aspartic proteases and β-1,3-1,4-glucanase [11]. It exhibits a broad growth temperature ranging from 25–55°C and the optimum growth temperature is at 50°C. *R. miehei* has industrial potentials due to its abilities of producing extracellular enzymes. To facilitate future investigations, we sequenced the genome of *R. miehei* CAU432. In combination with transcriptome analysis, the mechanisms of thermophilic adaptation in *R. miehei* were investigated for developing potential strategies to massively produce thermostable enzymes.

## Results

### General features of the genome

**Figure 1:**
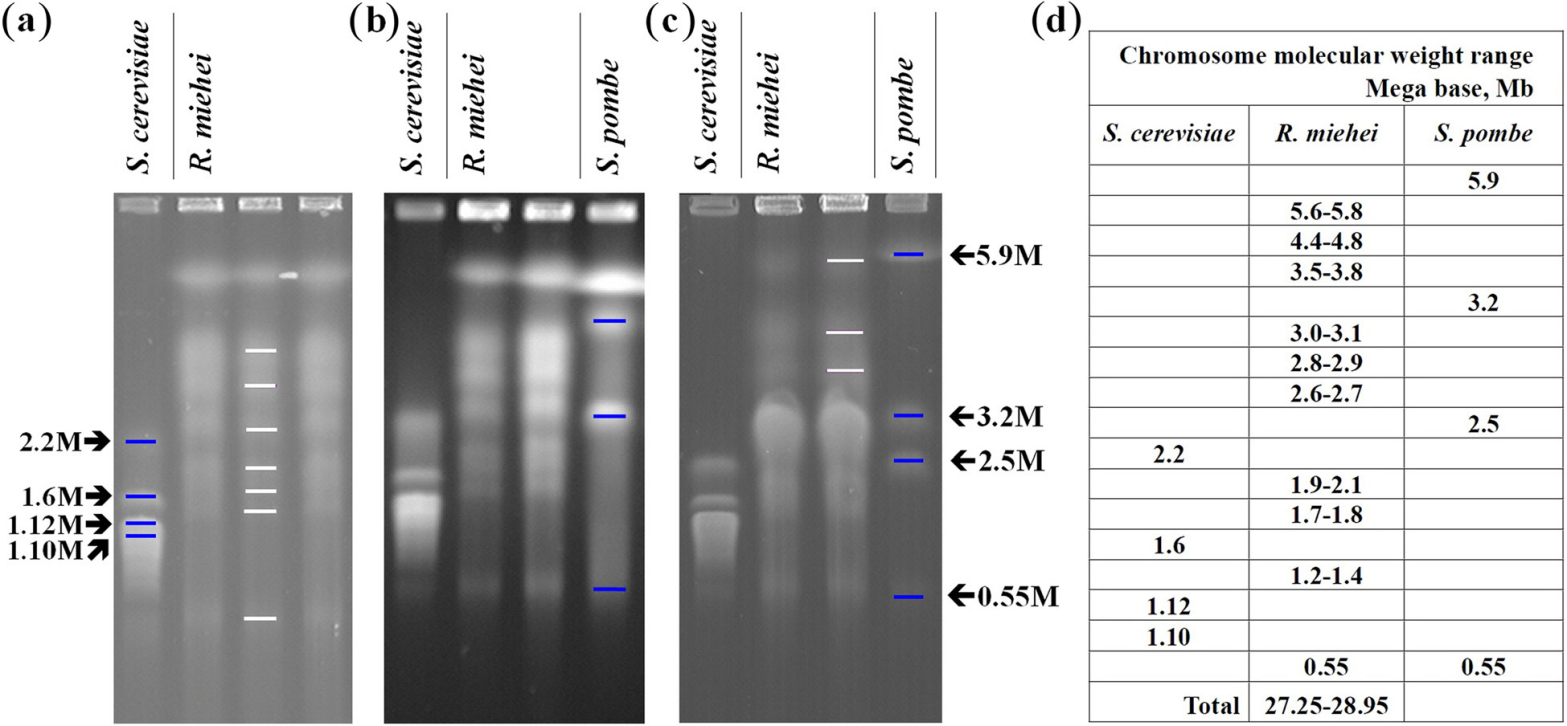
**Pulsed field gel electrophoresis (PFGE) analysis of the chromosome pattern of *R. miehei *CAU432.** Dark bands indicate the chromosome markers of *Saccharomyces cerevisiae* and *S. pombe*. White bands indicate the group bands of *R. miehei* chromosomes. The gel was electrophoresed under three different conditions: **(a)** Full set chromosome electrophoresis for 96 h with a switch interval ramp from 20 min to 40 min. **(b)** The middle range chromosome separation, gel electrophoresis for 60 h with a switch interval ramp from 20 min to 30 min, followed by 36 h with a switch interval ramp from 30 min to 40 min. **(c)** Large chromosomes electrophoresis for 96 h with a switch interval ramp from 40 min to 50 min. **(d)** The summary of estimated chromosome band molecular sizes on the PFGE gel.

Using a combination of Roche 454 and Illumina systems (in the Additional file 1: Table S1), the genome of *R. miehei* was shot-gun sequenced. The final assembly genome size is 27.6 Mb, N50 contig length is approximately 188.9 kb and N90 contig is in 61.4 kb. Gene prediction was performed using different models, which yielded 10,345 protein-coding genes with an average length of 1,621 bp (Table 1**)**. Total size of the genome is consistent with the estimation of approximately 27 to 29 Mb from 10 separated chromosomes between 0.55 to 5.8 Mb by pulsed field gel electrophoresis (PFGE) (Figure 1). The *R. miehei* genome (27.6 Mb) falls within the size of most available fungal genomes that has a range of 10–60 Mb with a median of 28 Mb (Table 2). This whole genome information has been deposited at DDBJ/EMBL/GenBank under the accession No. AGBC00000000.

**Table 1:** **Properties of the *R. miehei *** **CAU432 genome**

**Genome**	**Value**
Scaffolds/contigs (≥ 500 bp)	471
Contig N50 (bp)	188,961
Contig N90 (bp)	61,458
Mean (bp)	19,849
Max (bp)	619,110
Nuclear genome assembly size (Mb^***a***^)	27.6
G + C content (%) overall	43.8
G + C content (%) coding	47.4
G + C content (%) in introns	38.12
Protein coding gene number	10,345
Mean gene length (bp)	1,621
Percent coding (%)	47.1
Exons	52,179
Mean number per gene	5.0
Mean length (bp)	249.23
Introns	41,834
Mean number per gene	4.462
Mean length (bp)	90.0
RNA (tRNA number)	136

^***a***^Mb, mega base pairs.

**Table 2:** **General features of some fungal genomes compared with that of *R. miehei***

**Organism**	**Size (Mb**^***a***^**)**	**No. gene (coding)**	**Percent coding (%)**	**G + C content (%)**	**Reference**
*Aspergillus fumigatus*	29.4	9,926	50.1	49.9	[16]
*A. nidulans*	30.1	10,701	58.8	50.3	http://www.aspgd.org/
*A. niger*	33.9	14,165	-	50.4	[21]
*Fusarium graminearum*	36.4	14,038	48.76	48.04	http://www.broadinstitute.org/
*Magnaporthe grisea*	37.9	11,109	40.5	51.6	http://www.broadinstitute.org/
*Mucor circinelloides*	36.6	11,719	36.5	34.4	http://genome.jgi-psf.org/
*Myceliophthora thermophila*	38.7	9,110	-	51.4	[23]
*Neurospora crassa*	40.0	12,188	36.3	48.2	http://www.ncbi.nlm.nih.gov/genome/
*Penicillium chrysogenum*	32.2	12,943	56.6	48.9	http://www.ncbi.nlm.nih.gov/genome/
*Phanerochaete chrysosporium*	29.9	11,777	45	57	[15]
*Phycomyces blakesleeanus*	53.9	16,528	32.4	33.2	http://genome.jgi-psf.org/
*Podospora anserina*	36	10,545	44.75	52.02	[17]
***Rhizomucor miehei***	**27.6**	**10,345**	**47.1**	**43.83**	**This work**
*Rhizopus oryzae*	46.1	17,459	39	35.6	[26]
*Thermomyces lanuginosus*	23.3	5,105	55.6	52.14	[20]
*Thielavia terrestris*	36.9	9,813	-	54.7	[23]
*Trichoderma reesei*	33.9	9,129	40.4	52	[22]

^***a***^Mb, mega base pairs.

The average G + C content of the genome is 43.8% which is higher than those of zygomycete fungi (average 35.3%) but lower than most ascomycete fungi including three thermophilic ascomycetes, *T. lanuginosus* (52.14%), *T. terrestris* (54.7%) and *M. thermophila* (51.4%) (Table 2) [20,23-25]. Since G:C pairs are more thermally stable, it is somewhat surprising that the G + C content of thermophilic *R. miehei* whole genome (43.8%) and its coding genes (47.4%) are close to those of the mesophilic ascomycetous fungi (Table 2). However, the values are significantly higher than those of the mesophilic zygomycetes such as *Rhizopus oryzae*, average 35.3% and 35% for whole genome and protein coding genes, respectively [26]. The majority of predicted protein-coding genes contain multiple exons, with an average of 5 exons per gene and an average exon length of 249.2 bp (Table 1). Introns are of typical length in fungi, averaging between 80 bp and 150 bp [24]. *R. miehei* genome contains 0.877 Mb of repetitive sequences representing 3.17% of the assembled genome (in the Additional file 1: Table S2), which is much lower than that of *R. oryzae* (34.77%) [26]. The repetitive sequences in the *R. miehei* genome consist of recognizable transposable elements (TEs) including short interspersed nuclear elements (SINEs), long interspersed nuclear elements (LINEs), long terminal repeat (LTR) elements and unclassified interspersed repeats.

**Figure 2:**
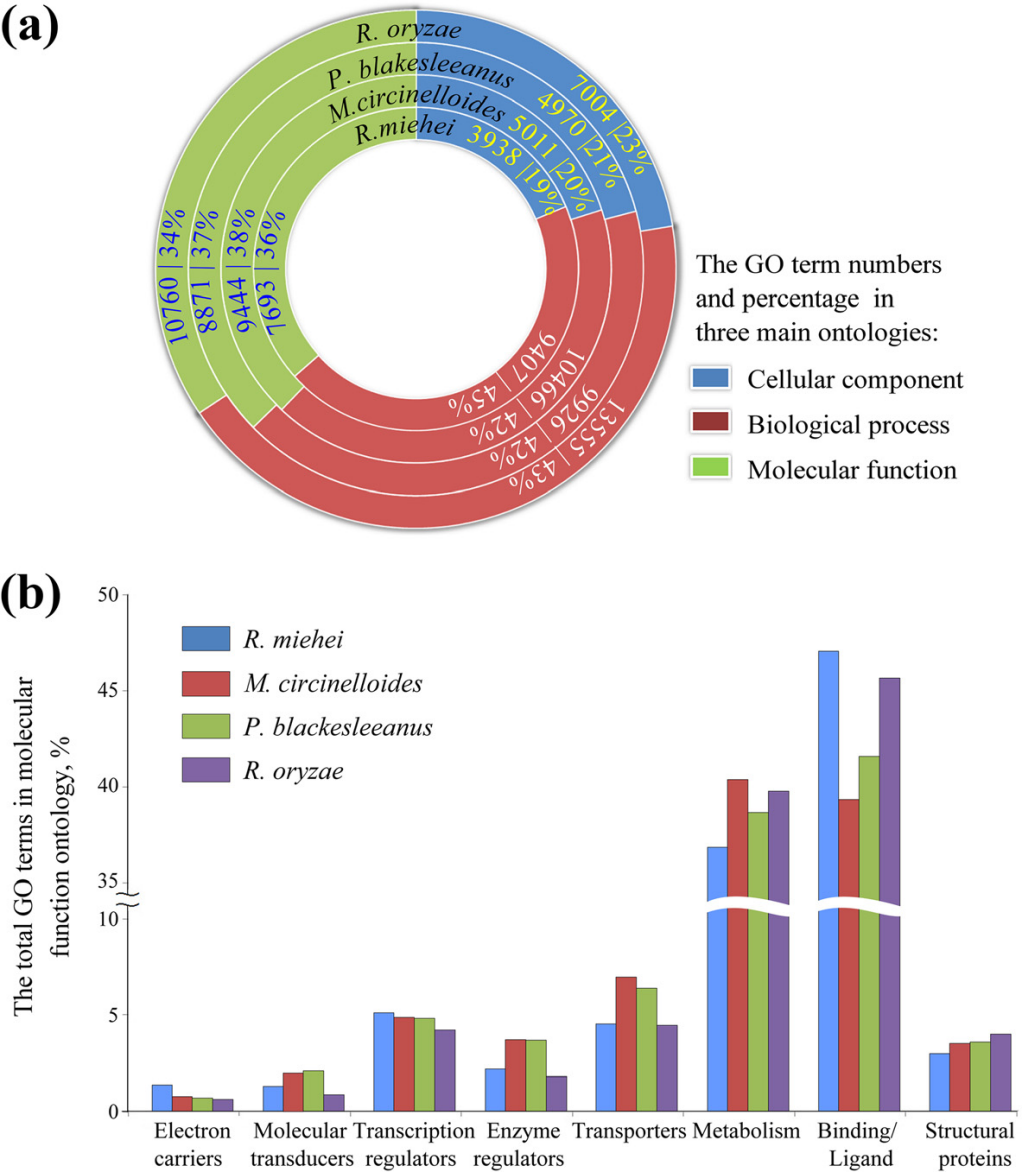
**Functional classifications of three zygomycete genomes compared with that of *R. miehei *CAU432. (a)** Comparison of the GO term numbers of *R. miehei*, *M. circinelloides, P. blakesleeanus* and *R. oryzae* in GO categories. Each circle represents the relative fraction of genes represented in each of the categories for each genome. The gene numbers are also shown. **(b)** The difference percentage of GO terms in molecular function category by gene ontology.

Of all predicted genes, 71.6% (7,407 genes) of the translated proteins show sequence similarity to proteins in the known databases, and 57.6% (5,961 genes) in *R. miehei* CAU432 can be annotated and attributed into different functional categories in Gene Ontology (GO) (Figure 2a). Compared to other three zygomycete fungi, *R. miehei* encodes a larger number of proteins with GO terms involved in protein binding group, electron carriers and transcription regulators in the molecular function category (Figure 2b).

### Phylogenetic and syntenic relationships

**Figure 3:**
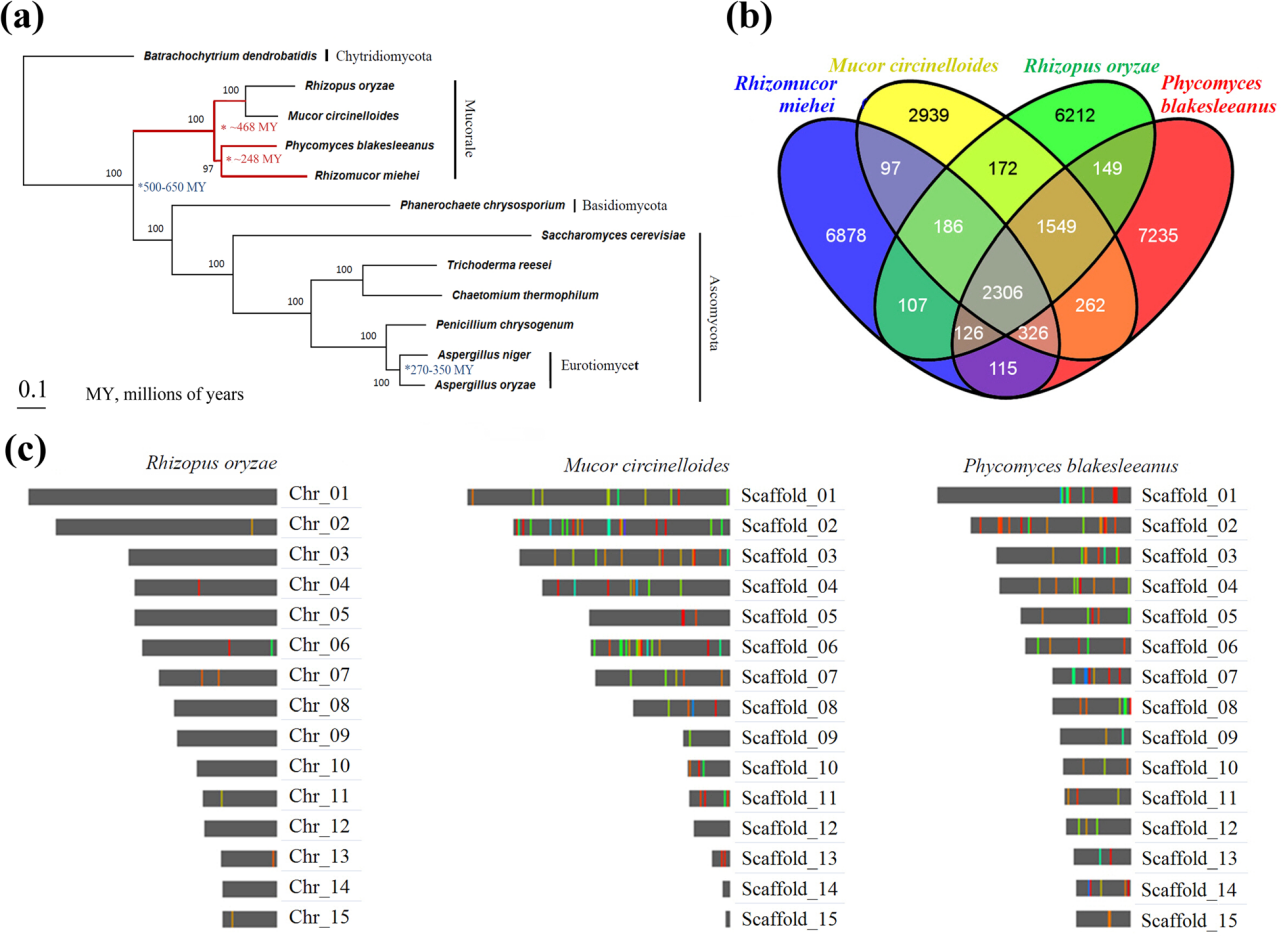
**Phylogenetic and syntenic relationship of *R. miehei *CAU432 among different fungi. (a)** Phylogenetic tree constructed using the Dayhoff amino acid substitution model showing the evolutionary relationships of 12 fungal species. Bar represents 0.1 substitutions per site. **(b)** Distribution of orthologues of *R. miehei, R. oryzae, M. circinelloides* and *P. blakesleeanus*. Venn diagram of the number of common genes with homologs in *R. miehei, R. oryzae, M. circinelloides* and *P. blakesleeanus*, showing the numbers of exclusive and common genes for different fungal genomes. **(c)** Distribution of the syntenic blocks of *R. miehei* in the 15 largest chromosomes/scaffolds of *R. oryzae, M. circinelloides* and *P. blakesleeanus*. The different synthetic blocks of *R. miehei* CAU432 were shown as different colour bands entrenched in the chromosomes/scallfolds.

The order of Mucorales consists of genera such as *Mucor*, *Phycomyces*, *Rhizopus*, and *Rhizomucor*. However, the phylogenetic relationships among the members of Mucoraceae and other fungi remain unclear. Based on a total of 409 orthologous protein sequences, a phylogenomic tree also placed *R. miehei* and *Phycomyces blakesleeanus* in the same clade while *R. oryzae* and *Mucor circinelloides* in another clade (Figure 3a). A phylogenomic analysis revealed that *R. miehei* and *P. blakesleeanus* diverged about 248 million years (MY) ago, and *R. miehei* diverged about 468 MY from *R. oryzae* and *M. circinelloides* (Figure 3a).

We found that there are 2,306 orthologues shared between *R. miehei* and other zygomycetes, *M. circinelloides*, *R. oryzae* and *P. blakesleeanus*. In addition to the common orthologues among *R. oryzae* and *P. blakesleeanus*, 441 (115 plus 326) shared orthologues were detected between *R. miehei* and *P. blakesleeanus* while only 293 (107 plus 186) between *R. miehei* and *R. oryzae* (Figure 3b). These numbers also suggest that *R. miehei* is closer to *P. blakesleeanus* than to *R. oryzae*. Syntenic relationship analysis of *R. miehei* with three other Mucorales indicated that a total of 169 syntenic blocks between *R. miehei* and *P. blakesleeanus* were detected while only 125 and 8 syntenic blocks were identified between *R. miehei* vs. *M. circinelloides* and *R. miehei* vs. *R. oryzae*, respectively (Figure 3c). These observations hence support the view that the genomic sequences of *R. miehei* are closer to *P. blakesleeanus* than to *M. circinelloides* and *R. oryzae*.

Our Pfam analysis identified 1,827 protein families (containing 3,979 proteins) in *R. miehei,* more than in *M. circinelloides* (1,804 families containing 4,682 proteins) and *R. oryzae* (1,683 families containing 6,076 proteins). A stochastic birth-and-death model [27] showed that relative to the most recent common ancestor of *R. oryzae* and *M. circinelloides*, 31 families including glycoside hydrolase (GH) families, phenol hydroxylase, metallopeptidase, lipase and transporter were expanded while 70 families (lipase, chitin synthase, Hsp70 protein, peptidase, transporter and transposase) were contracted in *R. miehei* (in the Additional file 1: Table S3).

### Carbohydrate-active enzymes in *Rhizomucor miehei* and other fungi

Carbohydrate-Active Enzymes (CAZys) participate in many important biological processes including cell wall synthesis, signaling and energy production. Our analysis found 110 glycoside hydrolases (GHs), 118 glycosyl transferases (GTs), 2 polysaccharide lyases (PLs), 20 carbohydrate esterases (CEs), and 20 carbohydrate binding modules (CBMs) in the genome of *R. miehei* (Table 3). Comparatively, lower numbers of GHs, PLs, CEs and CBMs were identified in *R. miehei* than in other fungi. In contrast, a higher number of GTs was identified (in the Additional file 1: Tables S4-S8). Comparative analysis identified CBM12, GH8, GH46, GT10, GT64 and GT77 unique to *R. miehei* except *R. oryzae *[26]. Relative to other fungi, the genome of *R. miehei* was particularly enriched with the GH families of GH15, GH31, GH36, GH37 and GH46, and GT families of GT2, GT15, GT49, GT62, GT64, and GT77. A CE family of CE4 and a PL family of PL14 were also found in the fungus (in the Additional file 1: Tables S4-S8).

**Table 3:** **Comparison of the number of CAZymes of *R. miehei *** **with those of other fungi**^***a***^

**Species**	**GH**	**GT**	**PL**	**CE**	**CBM**	**Total**
*Aspergillus nidulans* FGSC A4	251	92	19	30	41	433
*A. niger* CBS 513.88	243	118	8	23	43	435
*A. oryzae* RIB40	294	116	21	30	34	495
*Leptosphaeria maculans* v23.1.3	225	95	19	34	51	424
*Magnaporthe grisea* 70-15	232	92	4	46	65	439
*Neurospora crassa* OR74A	174	76	3	22	42	317
*Penicillium chrysogenum* Wisconsin_54-1255	216	102	9	22	49	398
*Phanerochaete chrysosporium*	182	66	4	16	48	316
*Podospora anserina* S mat+	226	87	7	41	97	458
***Rhizomucor miehei *** **CAU432**	**110**	**118**	**2**	**20**	**20**	**270**
*Rhizopus oryzae* RA 99-880	118	143	6	39	27	333

^***a***^Enzymes abbreviated based on CAZyme classification. GH, glycoside hydrolase; GT, glycosyl transferase; PL, polysaccharide lyase; CE, carbohydrate esterase; CBM, carbohydrate-binding module.

### Proteases

Proteolysis is ubiquitous in fungi, and is essential for protein degradation, amino acid assimilation and cellular differentiation. A total of 155 proteases (proteolytic enzymes or proteinases) were found in *R. miehei* CAU432, including 140 peptidases and/or proteases, and 15 proteosome and ubiquitin related proteases. *R. miehei* contains a unique collection of proteases (in the Additional file 2: Figure S1, in the Additional file 1: Table S9). Forty three of 155 predicted peptidases contain secreted signal peptides. Comparatively, the number of peptidases in the *R. miehei* genome is slightly higher than that of previously reported for *A. fumigatus* (136 peptidases), *A. oryzae* (134 peptidases) [28] and *T. terrestris* (143 peptidases) [23], but lower than that of *A. nidulans* (210 peptidases) and *A. niger* (169 peptidases). Also, the number of secreted peptidases is higher than that of other thermophilic fungi and fungal pathogens [18,23].

### Lipases and esterases

Lipases (triacylglycerol acylhydralases; EC 3.1.1.3) and esterases (carboxylic ester hydrolases; EC 3.1.1.1) collectively known as lipolytic enzymes are important industrial enzymes for biocatalysis, biorefining, food processing, the chiralspecific synthesis of pharmaceuticals and fungal toxin degradation due to their ability to catalyze many different reactions based on hydrolysis and synthesis of esters formed from glycerol and long-chain fatty acids [8,13,29,30]. The *R. miehei* genome contains a diverse array of genes that encode ester bond hydrolysis enzymes. Ninety seven genes belong to lipases, phospholipase and thiolester hydrolases (EC 3.1.-. -) while only 40 genes are glycerol ester hydrolases including the main group of “true” lipases, triacylglycerol lipase. Twenty nine genes belong to “true” phospholipase genes, lysophospholipase (EC 3.1.1.5) and 24 genes being thiolester and sulfuric-ester hydrolases (EC 3.1.2/6.-). Other ester bonds hydrolases are phosphorus-containing anhydrides (including other part of EC 3.1.- and all EC 3.6.-) consisting of 157 genes in total. There are 43 protein phosphatase genes in *R. miehei* CAU432 genome. The main type protein phosphatase genes are serine/threonine protein phosphatase and protein tyrosine phosphatase (EC 3.1.3.-).

### Cellulolytic enzymes

Cellulose degradation is achieved through the synergistic action of cellulases (endoglucanases), cellobiohydrolases (exoglucanases) and cellobiosidases (β-glucosidases). Specifically, there are four endoglucanases and eight β-glucosidases in the genome. Fungal cellobiohydrolases are classified in the families of GH6 and GH7 while β-glucosidases constitutes a major group among glycoside-hydrolyzing enzymes belonging to families GH1 and GH3. No genes belonging to families GH1, GH6 and GH7 were found in the *R. miehei* genome. Only eight GH3 family genes were identified in *R. miehei* in contrast to those filamentous ascomycetes typically containing more than ten genes (in the Additional file 1: Table S4 ). Otherwise, the *R. miehei* genome contains two putative endoglucanases (typical cellulose-degrading enzymes) assigned to the family GH45, which carry the sole CBM1 module. We also found two putative GH9 proteins (in the Additional file 1: Table S4), a family of endoglucanases mostly found in bacteria, plants, and occasionally in animals [31]. GH9 members are absent in most of ascomycete filamentous fungi but are also found in *R. oryzae* (in the Additional file 1: Table S4). The role of fungal GH9 remains unclear, but is probably unrelated to cellulose degradation [32]. Two mannan-degrading enzymes in family GH5 may be responsible for mannan digestion. A new glycoside hydrolase was identified in *R. miehei,* which belongs to the family GH125 of exo-α-1,6-mannosidases that contains one member from *Streptococcus pneumoniae* (SpGH125) and the other one from *Clostridium perfringens* (CpGH125) [33].

### Transcriptomes of the *R. miehei* CAU432

The transcriptional profiles of *R. miehei* CAU432 grown at two different temperatures (30°C and 50°C) were investigated by RNA-seq analysis (in the Additional file 2: Figure S2a). Approximately 35 million high-quality reads were generated from each library, and 62% reads were uniquely mapped to the genome (in the Additional file 2: Figure S2a). A high percentage of reads (67.1%) were mapped to predicted protein-coding genes, while 3.96% reads were distributed in splice junctions, 3.49% reads in antisense transcripts, 2.27% in introns, and 21.9% in other genomic regions (in the Additional file 2: Figure S2b). The frequency of reads which mapped to different genes ranged from one to over 300,000 (in the Additional file 2: Figure S2a and S2c).

The expression profiles of *R. miehei* at 30°C and 50°C were compared using the DEGseq program (in the Additional file 2: Figure S2d). More than 90% of predicted genes (9,680, 93.0% at 30°C; 9,618, 93.6% at 50°C) were detected with at least one read, while 128 genes and 190 genes were uniquely expressed at 50°C and 30°C, respectively (in the Additional file 2: Figure S3a). The results show that 2,117 genes were differently expressed (*P* < 0.001) by the fungus with more than two-fold changes (in the Additional file 2: Figure S3b and Figure S4). There were 849 up-regulated and 1,268 down-regulated genes in mycelia grown at 50°C (in the Additional file 2: Figure S4). These genes included ribosomal proteins, heat shock proteins, translation elongation factors, which involved in the protein synthesis. In contrast, the expression of proteasome subunits associated with the degradation of proteins decreased significantly (in the Additional file 2: Figure S4). These observations implicate that both increasing the production and decreasing the degradation of proteins can be further explored for industrial usage for *R. miehei* mycelia grown at 50°C. Many genes of the GHs, lipases and phospholipases were expressed at low expression levels in the mycelia grown at both 30°C and 50°C (Additional file 2: Figure S3b). Furthermore, most of the proteolytic enzymes exhibited a decreased expression level when the growth was shifted from 30°C to 50°C (in the Additional file 2: Figure S3b). These differently expressed genes fall into 31 Gene Ontology groups (in the Additional file 2: Figure S5). Of note, more up-regulated genes were annotated with GO terms of macromolecular complex and organelle part groups in cellular component category when the cells grew at 50°C compared to cells grown at 30°C.

## Discussion

We report here a genomic analysis of *R. miehei*, one of the most widely used extracellular enzyme producers. The phylum Zygomycota contains approximately ~900 species of true fungi. So far, over a hundred of fungal genomes have been sequenced and are publicly available. However, Zygomycetes have been rarely investigated both genetically and genomically. Results from this study suggest that *R. miehei* is closer to *P. blakesleeanus* than to *M. circinelloides* and *R. oryzae*, although *Rhizomucor*, *Mucor* and *Rhizopus* are classified under the same family Mucoraceae. *R. miehei* is well known for its capacity to produce large amounts of hydrolytic enzymes such as protease, lipase in industry. Here, we focus on a potential reservoir of thermostable enzymes from *R. miehei*.

Complete degradation of starch requires the combined action of three main types of amylases: α-amylase, glucoamylase and α-glucosidase. It is known that GH13 (including EC 3.2.1.1 α-amylase), GH15 (including EC 3.2.1.3 glucoamylase) and GH31 (including EC 3.2.1.20 α-glucosidase) are involved in amylolysis [34]. All three main amylases are present in multiple copies in the *R. miehei* genome. Specifically, there are six GH13, seven GH15 and eight GH31 enzymes identified in the genome. Of note, *R. miehei* contains the higher number (seven) of family GH15 enzymes for starch degradation, compared to the other fungal genomes (in the Additional file 1: Table S4).

Chitinolytic enzymes can be divided into chitinases (EC 3.2.1.14) and *N*-acetylglucosaminidases (EC 3.2.1.52). Chitinases are members of families GH18 and GH19. N-acetylglucosaminidases belong to families GH3 and GH20. The genomes of filamentous fungi typically contain between 10 and 25 different chitinases [23,35]. In *R. miehei*, a total of 13 GH18 genes and three GH20 genes were found, a value similar to what is found in known ascomycete and basidiomycete species (in the Additional file 1: Table S4). A gene of GH18 (RhzM07326) contains three CBM5 which has been found in bacterial enzymes and may bound weakly to several crystalline polysaccharides, while another one (RhzM00912) contains one CBM19 with chitin-binding function (in the Additional file 1: Table S7). Three genes belonging to GH20 are identified in the *R. miehei* genome and are possibly related to β-*N*-acetylhexosaminidases (EC 3.2.1.52). Unlike dikaryotic fungi, the cell wall of Mucorales usually contains a high percentage of chitin and chitosan which are synthesized by chitin synthases and chitin deacetylases [26]. Indeed, there are evidences that chitin and chitosan are an integral part of the cell wall structure and chitosan is a component of mycelia in *R. miehei *[36]. Family CE9 which includes *N*-acetylglucosamine-6-phosphate deacetylases (EC 3.5.1.25) is important for the metabolism of chitin. *R. miehei* contains one CE9 member (in the Additional file 1: Table S7), similar to ascomycete and basidiomycete fungi. Chitin deacetylase (EC 3.5.1.41) as one of the members of family CE4, hydrolyzes the acetamido group in the *N*-acetylglucosamine units of chitin and chitosan, leading to the production of glucosamine units and acetic acid. In *R. miehei*, a very large set of chitin deacetylases of family CE4 is found (in the Additional file 1: Table S7). Most of the previously reported chitin deacetylases often exist in multiple isoforms [37]. A total of 16 CE4 genes were found in *R. miehei* which is only second to the previous highest number of CE4 observed in the *Rhizopus oryzae *[26].

In the *R. miehei* genome, there was a high number of serine endopeptidases (EC 3.4.21.-), aspartic endopeptidases (EC 3.4.23.-), metalloendopeptidases (EC 3.4.24.-), and threonine endopeptidases (EC 3.4.25.1) which belongs to proteasome proteolytic subunits. For omega peptidases, only two ubiquitin-specific peptidases (EC 3.4.19.12) were found. As *Aspergillus oryzae* is known to have prominent potential for the secretory production of various enzymes [38], comparison of peptidase gene numbers between *R. miehei* and *A. oryzae* is shown in the Additional file 1: Table S9. The gene numbers of 36 serine endopeptidases, 18 aspartic endopeptidases, 33 metalloendopeptidases and 14 threonine endopeptidases are strikingly larger than those for *A. oryzae* (in the Additional file 1: Table S9). The large number of predicted peptidase genes is consistent with previous studies describing *R. miehei* as one of the best protease producers among the fungi [7,9]. *R. miehei* CAU432 has 18 genes encoding aspartic protease, only one (RhzM08045) has been so far cloned, characterized and has reached commercial product (Accession no. M18411) [36]. The aspartic proteinase from *R. miehei* (RMP) has a very high thermal stability, which is probably due to the high level of glycosylation [39]. *N*-linked carbohydrates enhance the thermal stability of glycoproteins. The corresponding gene has 2 *N*-glycosylation sites. Actually, most of other aspartic proteinase genes from *R. miehei* possess also multiple *N*-glycosylation sites. Thus, various aspartic proteinases from *R. miehei* may exhibit excellently thermal stability among aspartic proteinases.

Lipases and esterases are water-soluble enzymes that hydrolyze ester bonds of water-insoluble substrates such as triglycerides, phospholipids, and cholesteryl esters. Fungal lipases show remarkable levels of activity and stability in non-aqueous environments and are widely used in the catalysis of unnatural reactions such as esterification and transesterification [39]. *R. miehei* is also known to be a good producer of lipolytic enzymes [6,8]. Although only one lipase gene which corresponds to the gene (RhzM08505) has been isolated and characterized from *R. miehei* (Accession no. B34959) [40] to date, there are 31 lipase genes in the genome (in the Additional file 2: Figure S6b). The number of lipases in the *R. miehei* genome (in the Additional file 2: Figure S1) is higher than those of most of fungi [23], and the number of secreted lipases (9 in *R. miehei*) is above the average found in other fungi [19]. Besides, *R. miehei* has a large number of phospholipase genes (21 genes), and the number of secreted phospholipases is higher than that of human fungal pathogens such as *Malassezia globosa, Malassezia restricta *[41].

Thermophilic fungi can grow at elevated temperatures above 50°C [3]. Thermotolerance is critical for *R. miehei* to grow at high temperature ranges characteristic of original ecological habitat niche. Heat shock proteins (HSPs), chaperones, ubiquitin and proteasome related misfolding protein degradation are highly related to heat stress response by regulating many cellular processes [42-44]. Over expression of HSP70 in *Trichoderma* was evidenced to enhance fungal resistance to heat and other abiotic stresses [45]. HSP60, belonging to the intercompartmental transport proteins is thought to be important in thermophily for thermophilic fungi, was found to be highly expressed in the mycelia of *R. miehei* CAU432 at 50°C. Chaperones can mediate nascent protein folding, which is fundamentally important from thermophilic bacteria to plant [46,47]. In the *R. miehei* genome, there are 122 chaperone genes including Heat shock protein families, DnaJ, GrpE, HSP factors, peptidyl-prolyl cis-trans isomerase and other specific chaperones.

Proteasome activator complex subunit 4, proteasome subunits and chaperone regulatory proteins were significantly down-regulated at the high growth temperature. F-Box/WD-box proteins, RING finger proteins and ankyrins, together with limited cullins, the positive regulators of SCF-dependent ubiquitylation and subsequent protein degradation [43,48], all showed an increased expression when the culture of *R. miehei* was shifted to the high temperature (in the Additional file 2: Figure S4). These observations suggest that the protein synthesis and fate play important roles for *R. miehei* CAU432 to grow under temperature stress. Two related protein modification systems (viz. neddylation and sumoylation), whose proteins were designated as ubiquitin-like proteins (UBLs) which play a role in DNA damage repair under heat stress [49,50]. However, UBL proteins in *R. miehei* remain at low level of expression at both growth conditions compared to chaperone/ubiquitin/proteasome systems in the genome. Apart from the HSPs and DnaJ chaperones, peptidyl-prolyl cis-trans isomerase, T-complex proteins, UBX domain-containing proteins, rotamases (FK506 binding protein) [51] and vacuolar transporter chaperones were highly induced at high temperature (in the Additional file 2: Figure S4).

Non-reducing disaccharide trehalose, having the unique property of stabilizing membranes and enzymes against drying and thermal denaturation, accumulates upon heat, cold or osmotic stress [52]. Trehalose-phosphatase is the enzyme in charge of final release of trehalose. In the *R. miehei* genome, three trehalose-phosphatases (EC 3.1.3.12) and three trehalose-6-phosphate synthases (EC 2.4.1.15) were identified. These genes were expressed at high levels when cells were grown at 50°C. Of the trehalose synthesis pathway genes in *R. miehei,* five trehalases were found and two of them (RhzM00608 and RhzM03618) expressed at high levels at 50°C. The trehalase genes of RhzM01862 and RhzM07090 showed stable expression at both low and high temperatures. Compared to *R. oryzae* which contains three trehalose related genes [26], the fact that *R. miehei* has many more genes expressed at high temperature, suggests that trehalose plays an important role in the growth of mycelia at 50°C.

## Conclusion

The draft genome and transcriptome of *R. miehei* were investigated for the first time. We developed the fundamental genomic and molecular resources for characterization of *R. miehei. R. miehei* as a thermophilic fungus contains a large collection of genes for degrading its natural substrates, for coping different ecological conditions, and for mediating various stress during thermophilic growth. Based on the existence of a large repertoire of amylolytic, proteolytic and lipolytic genes in the genome, *R. miehei* has potential in the production of a variety of such enzymes.

## Methods

### Strains and genomic DNA preparation

*R. miehei* CAU432 used in the study (deposited at China General Microbiological Culture Collection Center under CGMCC No. 4967) was isolated from a pile of high-temperature hay due to anaerobic respiration in Sanmenxia city of Henan province, China. *R. miehei* CAU432 was maintained on potato dextrose-agar (PDA) plate as described by Tang et al. [11]. The strain was grown in rich medium (2% oat flour, 1% tryptone, 1% yeast extract, 5% KH_2_PO_4_, 0.3% MgSO_4_ · 7H_2_O, 0.3% CaCl_2_) at 50°C for 2 days in a shaker with a rotation speed of 200 rpm. Genomic DNA of *R. miehei* CAU432 was prepared from mycelia disrupted with pestle and mortar in liquid nitrogen. The genomic DNA was further purified by phenol–chloroform extraction as described by Sambrook and Russell [53].

### Pulsed field gel electrophoresis (PFGE)

Chromosomal DNA of *R. miehei* CAU432 was prepared as described by Orbach et al. [54]. Sporangiospores of *R. miehei* CAU432 obtained from young slant cultures were germinated in a complete medium (containing 0.1% yeast extract, 0.5% tryptone, 1% glucose) at 43°C, followed with isolation of germline protoplasts for chromosome analysis. Chromosomal DNA was prepared in agarose plugs with the CHEF Genomic DNA Plug kit (BioRad) following the instructions of the manufacturer. A 0.6% agarose gel in 0.5 × modified TBE (0.1 M Tris, 0.1 M Boric acid, 0.2 mM EDTA) was used to separate the chromosomes. Chromosome gel electrophoresis of contour-clamped homogeneous electric field (CHEF) was performed using the CHEF Mapper® XA Pulsed Field Electrophoresis System (Bio-Rad) in 0.6% UltraPure™ agarose (Invitrogen) gels at 16°C in circulating 0.5 × TBE buffer, 1.5 V/cm with different pulse intervals for 96 h. After separation, gels were stained in 0.5 μg/ml ethidium bromide for 1 h and then photographed under UV illumination.

### Genome sequencing and assembly

The sequencing of the genome of *R. miehei* CAU432 was performed using single-end reads from a fragment library and also using pair-end reads from two mate-pair libraries. The fragment library was sequenced according to Roche GS FLX Titanium Sequencing Method Manual. Approximately 16× genome coverage was obtained from fragment library. Two mate-pair libraries with insertion sizes of 2–3 kb and 6–10 kb were constructed and sequenced according to the manufacturer’s instructions (Illumina).

All of Roche 454 reads were assembled into 5,730 contigs using the software Newbler 2.3. The pair-end (both termini from the same DNA fragment) reads from 2–3 kb and 6–10 kb libraries were used to connect the assembled contigs, starting with short insert library and followed by iterating the scaffolding process, using longer insert library. A total of 1,522 intra-scaffold gaps were filled using the paired-end information to retrieve 454 reads whose ends were well-aligned on the adjacent contigs.

### Genome annotation and analysis

The tRNAs were identified using the software tRNAscan-SE-1.23. rRNAs were detected by using the software RNAmmer-1.2. Repetitive sequences in the *R. miehei* genome were identified by using RepeatModeler-1.0.4(http://www.repeatmasker.org/RepeatModeler.html) and were classified according to the classification criteria in the RepBase database (http://www.girinst.org/repbase/index.html). The resulting 38 families of repeats were added into the search library in RepeatMasker to find the repetitive sequences in the *R. miehei* genome [55].

ORFs were firstly predicted on all contigs by MAKER-2.10 software which combines the information of the gene structure based on the results produced from the TopHat software that maps RNA-seq reads against genomic sequences of *R. miehei* CAU432, with the gene structure information predicted from AUGUSTUS [56] (trained on sequences of *Rhizopus oryzae*) and GeneMark-ES version 1.0. Secondly, genes were selected only if the predicted protein lengths were more than 50 amino acids or 30 amino acids with BLAST evidence. Thirdly, for the regions without any MAKER prediction, non-overlapping gene models (supported with blast hits of ≥ 60% average identity and ≥80% query coverage) were picked; otherwise, the GeneMark-ES prediction was preferred. The final predictions of protein-coding genes were checked individually according to the BLAST evidence. Predicted genes were named as the abbreviated organism (RhzM) followed by a five-digit number. All the protein-coding genes were functionally assigned by homology to annotated genes from the NCBI nonredundant database, and Uniprot database and classified according to Gene Ontology (GO) and KEGG metabolic pathways. Secretory proteins were predicted by SignalP 3.0 Server at http://www.cbs.dtu.dk/services/SignalP/. Membrane proteins were predicted by TMHMM Server v. 2.0 at http://www.cbs.dtu.dk/services/TMHMM/.

### Construction of phylogenetic tree and detection of synteny blocks

Ortholog conservation in fungi was characterized with by OrthoMCL [57]. In total, 409 orthologous proteins were acquired and aligned with MUSCLE v3.8.31 [58]. A maximum likelihood phylogenomic tree was created using the concatenated amino acid sequences with the program TREE-PUZZLE using the Dayhoff model [59]. The divergence time between species was estimated with the Langley-Fitch method with r8s [60] by calibrating against the reassessed origins of the Ascomycota and Eurotiomycetes (at 500–650 and 270–350 million years (MY) ago, respectively) [61]. The protein families of the 12 fungi were identified by Pfam analysis with script pfam_scan.pl (ftp://ftp.sanger.ac.uk/pub/databases/Pfam/Tools) against the Pfam database (ftp://ftp.sanger.ac.uk/pub/databases/Pfam/current_release) [62]. The gene expansion and extraction of *R. miehei* relative to the most recent common ancestor of *R. oryzae* and *M. circinelloides* was analyzed with CAFE software using a stochastic birth and death model [27]. Single copy of orthologous groups of *R. miehei* were used to detect syntenic blocks among *R. miehei*, *R. oryzae* and *M. circinelloides* using the software OrthoCluster [63].

### Identification of carbohydrate-active enzymes in fungal proteomes

Annotation of carbohydrate-active genes was performed using the CAZy annotation pipeline [64]. The identification of genes that code for carbohydrate-active enzymes (GH, Glycoside Hydrolase; GT, glycosyltransferase; PL, polysaccharide lyase and CE, carbohydrate esterase) and their associated carbohydrate-binding module (CBM) in fungi was performed using CAZy database (http://www.cazy.org). Briefly, sequences of the proteins in CAZy were first divided by their constitutive modules (catalytic modules, carbohydrate binding module and other noncatalytic modules). Each *R. miehei* protein sequence was compared by the BLAST analysis against the library of around 50,000 individual modules. Protein sequences with an e-value < 0.1 were individually analyzed by multiple sequence alignment and search of conserved signatures/motifs. Each protein was also compared to a library of experimentally characterized proteins found in CAZy for functional description.

### Transcriptome analysis

For isolation of RNA, 50 ml of fresh rich medium were inoculated with 1% (50°C) or 5% (30°C) of 2 days old seeds to uniform the growth phase. Mycelia of *R. miehei* CAU432 after incubation at 30°C or 50°C for 1 day were ground to fine powder with liquid nitrogen prior to extraction. Total RNA was extracted using the Trizol reagent (Invitrogen, Carlsbad, US) and poly(A) mRNA was purified on oligo d(T) conjugated magnetic beads using FastTrack MAG mRNA Isolation Kits (Invitrogen, USA) according to the manufacturer’s protocol. The purified mRNA was fragmented, ligated with SOLiD™ adaptor mix and reversely transcribed by using SOLiD™ Total RNA-Seq Kit according to the manufacturer’s protocol. The constructed RNA-seq libraries were subjected to single end sequencing using Applied Biosystems SOLiD sequencing platforms. High quality reads were obtained after removing raw SOLiD reads with median QV (quality value) below 13.

Forty-five base pair (bp) long sequences were obtained from the termini of each exon read to construct the splice junction database. Fifty bp long RNA-seq reads were mapped to *R. miehei* assembled genome with the software Corona_Lite_Plus_4.2.1 (Applied Biosystems) at 5 mismatches while the unmapped reads were mapped to the splice junction database. In the order of first 45 bp (at 4 mismatches), 40 bp (at 4 mismatches), 35 bp (at 3 mismatches) and 30 bp (at 3 mismatches) sequences obtained from unmapped reads were iteratively mapped to genome and splice junction database. Reads with multiple alignments were discarded. Differentially expressed gene under two growth temperatures were identified using the DEGseq software [65] by the following criteria: *P* < 0.001 and normalized fold change of comparing two temperatures being greater more than 1 or less than -1.

### Availability of supporting data

The *Rhizomucor miehei* CAU432 whole genome sequence has been submitted to GenBank and deposited at DDBJ/EMBL/GenBank under the accession No. AGBC00000000 (http://www.ncbi.nlm.nih.gov/nuccore/AGBC00000000). The version described in this paper is the first version, AGBC01000000. The RNA-seq data have been deposited at NCBI Short Read Archive under series accession No. SRP008125 (http://www.ncbi.nlm.nih.gov/sra/?term=SRP008125) for the samples of *R. miehei* CAU432 growing at 30°C and 50°C, respectively. The sequence and annotation data are also available at http://foodenzyme.cau.edu.cn/data_base/index.html. These include genome sequence, datasets for genes and proteins, a summary of the results from Pfam analyses and a Blast server.

## Competing interests

The authors declare that they have no competing interests.

## Authors’ contributions

BZ, GZ and ZD assembled and finished the genome sequence, processed the raw data and performed shotgun assembly and contig scaffolding, manually curated the computer-generated annotation, and analyzed the annotation. PZ and YT isolated RNA and DNA, performed the 454/Roche sequencing and made libraries and prepared Figures 1, 2 and 3. SC performed all post-shotgun assembly bioinformatics aspects of the study, assisted in gap closure and in determining sequence accuracy. QY, SY and CC wrote parts of the main manuscript. BH participated and supervised the CAZy annotation. ZJ designed and coordinated the study, and wrote parts of the manuscript. All authors read and approved the final manuscript.

## Supplementary Material

Additional file 1: Table S1 Libraries used for the *R. miehei* CAU432 genome sequencing. **Table S2.** Transposable Elements (TEs) in the *R. miehei* CAU432 genome. **Table S3.** Expanded (highlighted in green) and contracted (highlighted in yellow) protein families in *R. miehei* CAU432. **Table S4.** Comparison of the number of GHs of *R. miehei* CAU432 with those of other fungi. **Table S5.** Comparison of the number of GTs of *R. miehei* CAU432 with those of other fungi. **Table S6.** Comparison of the number of PLs of *R. miehei* CAU432 with those of other fungi. **Table S7.** Comparison of the number of CEs of *R. miehei* CAU432 with those of other fungi. **Table S8.** Comparison of the number of CBMs of *R. miehei* CAU432 with those of other fungi. **Table S9.** Numbers of peptidase genes in *R. miehei* and *Aspergillus oryzae*. Click here for file

Additional file 2: Figure S1 Location of various enzymes in *R. miehei* CAU432. **Figure S2.** RNA-seq analysis of *R. miehei* transcriptome from mycelia growing at 30°C and 50°C. **Figure S3.** Gene expression in *R. miehei* CAU432 mycelia grown at 30°C (labeled as T30) and 50°C (labeled as T50). **Figure S4.** Gene expression changes in mycelia of *R. miehei* CAU432 growth at 50°C comparing with that of growth at 30°C. **Figure S5.** Molecular functions of Gene Ontology for differently expressed genes with at least two-fold changes between two growth temperatures in *R. miehei* CAU432. Click here for file
