# Supplementary material for: Genome sequence and transcriptome analyses of the thermophilic zygomycete fungus Rhizomucor miehei
Source: BMC Genomics. 2014 Apr 21;15:294. doi: 10.1186/1471-2164-15-294 (PMC4023604; doi:10.1186/1471-2164-15-294)
Supplement: Additional file 1: Table S1 — Libraries used for the R. miehei CAU432 genome sequencing. Table S2. Transposable Elements (TEs) in the R. miehei CAU432 genome. Table S3. Expanded (highlighted in green) and contracted (highlighted in yellow) protein families in R. miehei CAU432. Table S4. Comparison of the number of GHs of R. miehei CAU432 with those of other fungi. Table S5. Comparison of the number of GTs of R. miehei CAU432 with those of other fungi. Table S6. Comparison of the number of PLs of R. miehei CAU432 with those of other fungi. Table S7. Comparison of the number of CEs of R. miehei CAU432 with those of other fungi. Table S8. Comparison of the number of CBMs of R. miehei CAU432 with those of other fungi. Table S9. Numbers of peptidase genes in R. miehei and Aspergillus oryzae. [file 1471-2164-15-294-S1.doc]

**Additional files**

**Table S1 Libraries used for the *R. miehei* CAU432 genome sequencing**

| Library designation | Insert size | Reads length (average) | No. reads | No. unique reads | Average coverage |
| --- | --- | --- | --- | --- | --- |
| 454 Fragment Lib | 500-800 bp | 372 bp | 1,569,975 | 1,459,840 | 16 |
| Solexa Lib1 | 2-3 kb | 80 bp | 67,883,934 | 33,363,178 | 96 |
| Solexa Lib2 | 6-10 kb | 80 bp | 42,221,530 | 21,992,471 | 63 |

**Table S2 Transposable Elements (TEs) in the *R. miehei* CAU432 genome**

|  | Number of elements*^a^* | Length occupied (bp) | Percentage of sequence (%) |
| --- | --- | --- | --- |
| SINEs*^b^* | 196 | 51,340 | 0.19 |
| LINEs*^b^* | 52 | 41,875 | 0.15 |
| LTR*^b^* elements | 39 | 34,020 | 0.12 |
| DNA elements | 570 | 210,468 | 0.76 |
| Unclassified | 2,178 | 539,339 | 1.95 |
| Total interspersed repeats |  | 877,042 | 3.17 |

*^a^* Most repeats fragmented by insertions or deletions have been counted as one element.

*^b^* LINE, long interspersed nuclear element; LTR, long terminal repeat; SINE, short interspersed nuclear element.

**Table S3 Expanded (highlighted in green) and contracted (highlighted in yellow) protein families in *R. miehei* CAU432**

| RMCA | R. mie | R. ory | M. cir | P-value | Pfam Desciription |
| --- | --- | --- | --- | --- | --- |
| 102 | 103 | 90 | 120 | 0 | Major Facilitator Superfamily |
| 51 | 53 | 54 | 47 | 0.045 | ATPase family associated with various cellular activities (AAA) |
| 16 | 18 | 16 | 16 | 0 | Aldehyde dehydrogenase family |
| 0 | 17 | 483 | 15 | 0 | Reverse transcriptase (RNA-dependent DNA polymerase) |
| 13 | 14 | 20 | 10 | 0 | Developmentally Regulated MAPK Interacting Protein |
| 11 | 12 | 9 | 17 | 0 | GDSL-like Lipase/Acylhydrolase |
| 9 | 10 | 10 | 8 | 0 | FAD binding domain |
| 4 | 10 | 6 | 4 | 0.006 | Putative transposase DNA-binding domain |
| 5 | 9 | 5 | 5 | 0.001 | Glycosyl hydrolases family 31 |
| 6 | 8 | 2 | 9 | 0.036 | GRAM domain |
| 5 | 7 | 6 | 5 | 0 | Glycosyl hydrolase family 3 C terminal domain |
| 5 | 7 | 6 | 5 | 0 | Glycosyl hydrolase family 3 N terminal domain |
| 4 | 7 | 8 | 3 | 0 | Domain of unknown function (DUF3425) |
| 5 | 6 | 3 | 6 | 0.013 | Carbon-nitrogen hydrolase |
| 3 | 5 | 2 | 6 | 0.043 | Anp1 |
| 4 | 5 | 3 | 5 | 0 | Protein of unknown function (DUF3712) |
| 4 | 5 | 3 | 4 | 0 | Melibiase |
| 3 | 4 | 3 | 3 | 0.045 | Ferric reductase NAD binding domain |
| 2 | 4 | 5 | 1 | 0 | CP2 transcription factor |
| 2 | 4 | 3 | 1 | 0.019 | Coprinus cinereus mating-type protein |
| 2 | 3 | 1 | 2 | 0 | Glutathione-dependent formaldehyde-activating enzyme |
| 0 | 3 | 0 | 0 | 0 | Phenol hydroxylase, C-terminal dimerisation domain |
| 1 | 3 | 1 | 1 | 0.03 | Glycosyl hydrolases family 35 |
| 1 | 2 | 1 | 1 | 0 | Fungalysin metallopeptidase (M36) |
| 1 | 2 | 4 | 0 | 0.042 | HOOK protein |
| 0 | 2 | 0 | 0 | 0.047 | Glycosyl hydrolase family 12 |
| 1 | 2 | 0 | 2 | 0.028 | Alpha-L-fucosidase |
| 1 | 2 | 0 | 3 | 0.017 | Phosphate transporter family |
| 1 | 2 | 3 | 0 | 0.043 | SH3-binding, glutamic acid-rich protein |
| 1 | 2 | 1 | 2 | 0.022 | Protein of unknown function (DUF1446) |
| 0 | 1 | 0 | 0 | 0.038 | Cryptococcal mannosyltransferase 1 |
| 64 | 48 | 64 | 63 | 0 | Mitochondrial carrier protein |
| 41 | 38 | 39 | 48 | 0 | Sugar (and other) transporter |
| 37 | 30 | 40 | 39 | 0.003 | E1-E2 ATPase |
| 33 | 28 | 35 | 36 | 0.002 | bZIP transcription factor |
| 37 | 28 | 39 | 37 | 0 | AMP-binding enzyme |
| 28 | 27 | 26 | 29 | 0 | Zinc-binding dehydrogenase |
| 28 | 24 | 32 | 29 | 0 | Transmembrane amino acid transporter protein |
| 31 | 23 | 35 | 28 | 0 | Eukaryotic aspartyl protease |
| 22 | 21 | 23 | 22 | 0 | PPR repeat |
| 30 | 21 | 26 | 41 | 0 | Acetyltransferase (GNAT) family |
| 45 | 17 | 237 | 8 | 0 | 'chromo' (CHRromatin Organisation MOdifier) domain |
| 21 | 16 | 22 | 21 | 0 | Hsp70 protein |
| 18 | 16 | 25 | 15 | 0.012 | Septin |
| 21 | 16 | 31 | 18 | 0 | RasGEF domain |
| 30 | 16 | 37 | 32 | 0 | Polysaccharide deacetylase |
| 21 | 15 | 15 | 25 | 0 | ABC transporter transmembrane region |
| 25 | 15 | 27 | 27 | 0 | Cyclin |
| 20 | 14 | 24 | 21 | 0 | Chitin synthase |
| 15 | 13 | 14 | 19 | 0.009 | Domain of unknown function DUF |
| 22 | 13 | 25 | 21 | 0 | Amino acid permease |
| 13 | 13 | 16 | 13 | 0.005 | Basic region leucine zipper |
| 14 | 13 | 15 | 15 | 0.002 | Glycolipid 2-alpha-mannosyltransferase |
| 17 | 12 | 18 | 18 | 0 | Lipase (class 3) |
| 14 | 12 | 15 | 15 | 0.003 | PA domain |
| 13 | 11 | 13 | 15 | 0.006 | Enoyl-CoA hydratase/isomerase family |
| 28 | 10 | 110 | 12 | 0 | Retrotransposon gag protein |
| 14 | 10 | 14 | 16 | 0.043 | Cation transporting ATPase, C-terminus |
| 11 | 9 | 12 | 12 | 0 | NmrA-like family |
| 12 | 8 | 16 | 11 | 0.044 | KOW motif |
| 11 | 8 | 12 | 10 | 0 | NAD dependent epimerase/dehydratase family |
| 10 | 8 | 12 | 9 | 0 | Sodium/calcium exchanger protein |
| 11 | 8 | 9 | 15 | 0.002 | Velvet factor |
| 11 | 7 | 14 | 9 | 0 | MYND finger |
| 11 | 7 | 10 | 11 | 0 | ABC-2 type transporter |
| 16 | 6 | 21 | 17 | 0 | CotH protein |
| 9 | 6 | 11 | 9 | 0.005 | Fasciclin domain |
| 6 | 5 | 6 | 10 | 0.02 | Protein of unknown function (DUF2422) |
| 8 | 5 | 6 | 9 | 0 | Taurine catabolism dioxygenase TauD, TfdA family |
| 6 | 5 | 7 | 6 | 0.003 | Protein of unknown function (DUF2421) |
| 10 | 5 | 12 | 9 | 0 | Male sterility protein |
| 8 | 5 | 8 | 8 | 0 | CDR ABC transporter |
| 9 | 5 | 9 | 10 | 0.001 | OPT oligopeptide transporter protein |
| 8 | 4 | 9 | 8 | 0.001 | Fatty acid hydroxylase superfamily |
| 6 | 4 | 526 | 3 | 0 | Transposase |
| 5 | 4 | 5 | 6 | 0 | IBR domain |
| 5 | 4 | 3 | 6 | 0 | SUR7/PalI family |
| 3 | 4 | 2 | 4 | 0.001 | Fumarylacetoacetate (FAA) hydrolase family |
| 3 | 4 | 4 | 3 | 0.01 | HAMP domain |
| 6 | 4 | 3 | 8 | 0 | Flavin-binding monooxygenase-like |
| 3 | 4 | 3 | 4 | 0.02 | CoA-transferase family III |
| 4 | 3 | 3 | 6 | 0.014 | Hsp20/alpha crystallin family |
| 6 | 3 | 6 | 6 | 0.001 | Peptidase family M20/M25/M40 |
| 4 | 3 | 4 | 6 | 0 | POT family |
| 4 | 3 | 7 | 3 | 0.002 | Phosphoesterase family |
| 5 | 3 | 7 | 4 | 0.004 | Protein of unknown function (DUF3759) |
| 12 | 3 | 24 | 7 | 0 | DDE superfamily endonuclease |
| 3 | 3 | 3 | 2 | 0 | Serine hydrolase (FSH1) |
| 12 | 2 | 32 | 1 | 0 | PIF1 helicase |
| 45 | 2 | 127 | 4 | 0 | Transposase family tnp2 |
| 6 | 2 | 5 | 10 | 0 | Isochorismatase family |
| 5 | 2 | 5 | 5 | 0.005 | Histidine acid phosphatase |
| 3 | 2 | 3 | 4 | 0 | Peroxidase |
| 11 | 2 | 14 | 12 | 0 | UDP-glucoronosyl and UDP-glucosyl transferase |
| 1 | 2 | 4 | 0 | 0.031 | Ribosomal L40e family |
| 2 | 1 | 1 | 2 | 0 | Phosphotransferase enzyme family |
| 2 | 1 | 1 | 2 | 0.002 | Epoxide hydrolase N terminus |
| 10 | 1 | 38 | 5 | 0 | Transcriptional activator of glycolytic enzymes |
| 3 | 1 | 30 | 1 | 0 | Aspartyl protease |
| 3 | 1 | 2 | 3 | 0.05 | DJ-1/PfpI family |
| 2 | 1 | 3 | 2 | 0.024 | UbiA prenyltransferase family |

Note: The protein family dataset of the most recent common ancestor of *R. oryzae* and *M. circinelloides* (MRCA of RM) was used as a reference for analysis. Fungal species are abbreviated as: M. cir, *Mucor circinelloides*; R. mie, *Rhizomucor miehei*; R. ory, *Rhizopus oryzae*.

**Table S4 Comparison of the number of GHs of *R. miehei* CAU432 with those of other fungi**

| GH | R. org | R. mie | P. chr | N. cra | P. ans | M. gri | A. org | A. nig | P. chr | A. nid | L. mac |
| --- | --- | --- | --- | --- | --- | --- | --- | --- | --- | --- | --- |
| 1 | 0 | **0** | 2 | 1 | 1 | 2 | 3 | 3 | 3 | 3 | 3 |
| 2 | 0 | **0** | 2 | 5 | 7 | 6 | 7 | 6 | 6 | 10 | 7 |
| 3 | 6 | **8** | 11 | 9 | 11 | 19 | 23 | 17 | 17 | 20 | 13 |
| 5 | 7 | **7** | 19 | 7 | 12 | 13 | 14 | 10 | 13 | 15 | 15 |
| 6 | 0 | **0** | 1 | 3 | 4 | 3 | 1 | 2 | 1 | 2 | 3 |
| 7 | 0 | **0** | 9 | 5 | 6 | 6 | 3 | 2 | 2 | 3 | 3 |
| 8 | 1 | **1** | 0 | 0 | 0 | 0 | 0 | 0 | 0 | 0 | 0 |
| 9 | 4 | **2** | 1 | 0 | 0 | 0 | 0 | 0 | 0 | 0 | 0 |
| 10 | 0 | **0** | 6 | 4 | 8 | 5 | 4 | 1 | 3 | 3 | 3 |
| 11 | 0 | **0** | 1 | 2 | 6 | 5 | 4 | 4 | 1 | 2 | 2 |
| 12 | 0 | **2** | 2 | 1 | 2 | 3 | 4 | 4 | 3 | 1 | 3 |
| 13 | 4 | **6** | 9 | 10 | 9 | 10 | 17 | 18 | 16 | 13 | 7 |
| 15 | 6 | **7** | 2 | 2 | 3 | 2 | 3 | 2 | 3 | 2 | 3 |
| 16 | 8 | **7** | 23 | 14 | 12 | 16 | 13 | 13 | 16 | 13 | 18 |
| 17 | 3 | **4** | 2 | 4 | 4 | 7 | 5 | 5 | 5 | 5 | 8 |
| 18 | 15 | **13** | 11 | 12 | 20 | 14 | 18 | 14 | 9 | 19 | 10 |
| 20 | 4 | **3** | 3 | 0 | 1 | 2 | 3 | 3 | 2 | 2 | 2 |
| 23 | 0 | **0** | 0 | 0 | 0 | 0 | 0 | 0 | 0 | 0 | 0 |
| 24 | 0 | **0** | 0 | 0 | 1 | 1 | 2 | 0 | 0 | 1 | 0 |
| 25 | 0 | **1** | 1 | 1 | 0 | 0 | 1 | 0 | 1 | 3 | 0 |
| 26 | 0 | **0** | 0 | 1 | 1 | 0 | 1 | 1 | 1 | 3 | 1 |
| 27 | 2 | **2** | 3 | 0 | 2 | 4 | 3 | 5 | 1 | 3 | 4 |
| 28 | 18 | **4** | 4 | 2 | 0 | 3 | 21 | 21 | 5 | 9 | 6 |
| 29 | 0 | **2** | 0 | 0 | 0 | 4 | 0 | 1 | 0 | 0 | 1 |
| 30 | 0 | **0** | 2 | 2 | 3 | 1 | 0 | 1 | 1 | 0 | 2 |
| 31 | 3 | **8** | 5 | 5 | 5 | 5 | 10 | 7 | 11 | 10 | 8 |
| 32 | 0 | **0** | 0 | 1 | 0 | 5 | 4 | 6 | 7 | 2 | 2 |
| 33 | 0 | **0** | 0 | 0 | 0 | 0 | 0 | 0 | 1 | 0 | 0 |
| 35 | 1 | **3** | 3 | 2 | 1 | 0 | 7 | 5 | 2 | 4 | 4 |
| 36 | 4 | **4** | 0 | 1 | 1 | 2 | 3 | 2 | 2 | 4 | 2 |
| 37 | 5 | **5** | 2 | 2 | 2 | 2 | 1 | 1 | 1 | 1 | 1 |
| 38 | 2 | **2** | 1 | 1 | 1 | 2 | 1 | 1 | 1 | 1 | 1 |
| 39 | 0 | **0** | 0 | 0 | 0 | 1 | 0 | 0 | 2 | 1 | 1 |
| 42 | 0 | **0** | 0 | 0 | 0 | 0 | 0 | 0 | 1 | 0 | 0 |
| 43 | 2 | **2** | 4 | 7 | 10 | 19 | 20 | 10 | 14 | 15 | 11 |
| 45 | 5 | **2** | 2 | 1 | 2 | 1 | 0 | 0 | 0 | 1 | 2 |
| 46 | 2 | **2** | 0 | 0 | 0 | 0 | 0 | 0 | 0 | 0 | 0 |
| 47 | 10 | **7** | 6 | 8 | 9 | 6 | 5 | 5 | 6 | 7 | 8 |
| 51 | 0 | **1** | 2 | 1 | 1 | 3 | 3 | 4 | 3 | 2 | 3 |
| 53 | 0 | **0** | 1 | 1 | 1 | 1 | 1 | 2 | 1 | 1 | 1 |
| 54 | 0 | **0** | 0 | 1 | 0 | 1 | 1 | 1 | 1 | 1 | 0 |
| 55 | 0 | **0** | 2 | 6 | 7 | 3 | 4 | 3 | 3 | 6 | 3 |
| 61 | 0 | **0** | 15 | 14 | 33 | 17 | 8 | 7 | 4 | 9 | 20 |
| 62 | 0 | **0** | 0 | 0 | 2 | 3 | 2 | 1 | 1 | 2 | 1 |
| 63 | 2 | **0** | 1 | 1 | 1 | 2 | 1 | 1 | 1 | 1 | 3 |
| 64 | 0 | **0** | 0 | 2 | 1 | 1 | 0 | 0 | 2 | 0 | 1 |
| 65 | 0 | **0** | 0 | 0 | 0 | 0 | 1 | 1 | 1 | 1 | 1 |
| 67 | 0 | **0** | 0 | 1 | 1 | 1 | 1 | 1 | 1 | 1 | 1 |
| 71 | 0 | **0** | 3 | 6 | 3 | 0 | 8 | 7 | 6 | 5 | 1 |
| 72 | 2 | **1** | 1 | 5 | 6 | 4 | 7 | 7 | 6 | 5 | 6 |
| 74 | 0 | **0** | 4 | 1 | 1 | 1 | 0 | 1 | 0 | 2 | 0 |
| 75 | 0 | **0** | 0 | 1 | 1 | 1 | 4 | 2 | 1 | 2 | 0 |
| 76 | 0 | **0** | 0 | 10 | 9 | 7 | 11 | 11 | 8 | 7 | 7 |
| 78 | 0 | **0** | 1 | 0 | 1 | 1 | 9 | 8 | 5 | 8 | 1 |
| 79 | 0 | **0** | 5 | 2 | 3 | 1 | 6 | 3 | 2 | 1 | 1 |
| 81 | 1 | **1** | 0 | 1 | 1 | 2 | 1 | 1 | 1 | 1 | 2 |
| 84 | 0 | **0** | 0 | 0 | 0 | 0 | 0 | 0 | 1 | 0 | 0 |
| 85 | 1 | **1** | 1 | 0 | 0 | 0 | 0 | 0 | 0 | 0 | 1 |
| 88 | 0 | **0** | 1 | 0 | 0 | 1 | 3 | 1 | 0 | 2 | 1 |
| 89 | 0 | **0** | 2 | 0 | 0 | 0 | 1 | 0 | 0 | 0 | 0 |
| 92 | 0 | **0** | 4 | 2 | 2 | 6 | 6 | 5 | 4 | 5 | 6 |
| 93 | 0 | **0** | 0 | 2 | 3 | 1 | 3 | 0 | 2 | 2 | 2 |
| 94 | 0 | **0** | 0 | 1 | 1 | 1 | 0 | 0 | 0 | 0 | 1 |
| 95 | 0 | **0** | 1 | 0 | 0 | 1 | 3 | 2 | 1 | 3 | 1 |
| 105 | 0 | **1** | 0 | 1 | 0 | 3 | 4 | 2 | 2 | 3 | 3 |
| 114 | 0 | **0** | 0 | 1 | 1 | 1 | 1 | 2 | 2 | 2 | 2 |
| 115 | 0 | **0** | 1 | 1 | 3 | 0 | 4 | 0 | 0 | 1 | 2 |
| 125 | 0 | **1** | 0 | 0 | 0 | 0 | 0 | 0 | 0 | 0 | 0 |

Note: *R. miehei* CAU432 appears in bold. Enzymes abbreviated based on CAZyme classification. GHs, glycoside hydrolases. Fungal species are abbreviated as: R. org, *Rhizopus oryzae* RA 99-880; *R. mie,* *Rhizomucor miehei* CAU432; P. chr, *Phanerochaete chrysosporium*; N. cra, *Neurospora crassa* OR74A; P. ans, *Podospora anserine* S mat+; M. gri, *Magnaporthe grisea* 70-15; A. org, *Aspergillus oryzae* RIB40; A. nig, *Aspergillus niger* CBS 513.88**;** P. chr, *Penicillium chrysogenum* Wisconsin 54-1255; A. nid, *Aspergillus nidulans* FGSC A4; L. mac, *Leptosphaeria maculans* v23.1.3.

**Table S5 Comparison of the number of GTs of *R. miehei* CAU432 with those of other fungi**

| GT | R. org | R. mie | P. chr | N. cra | P. ans | M. gri | A. org | A. nig | P. chr | A. nid | L. mac |
| --- | --- | --- | --- | --- | --- | --- | --- | --- | --- | --- | --- |
| 1 | 15 | **5** | 6 | 6 | 8 | 12 | 9 | 12 | 6 | 9 | 6 |
| 2 | 35 | **27** | 13 | 11 | 16 | 8 | 18 | 18 | 13 | 12 | 16 |
| 3 | 3 | **1** | 1 | 1 | 1 | 1 | 1 | 1 | 1 | 1 | 1 |
| 4 | 6 | **7** | 4 | 6 | 5 | 4 | 9 | 8 | 5 | 7 | 5 |
| 5 | 2 | **2** | 1 | 2 | 2 | 1 | 3 | 5 | 4 | 2 | 0 |
| 8 | 4 | **3** | 9 | 2 | 3 | 2 | 4 | 5 | 6 | 5 | 9 |
| 10 | 1 | **2** | 0 | 0 | 0 | 0 | 0 | 0 | 0 | 0 | 0 |
| 15 | 14 | **13** | 2 | 5 | 5 | 4 | 3 | 3 | 3 | 3 | 3 |
| 17 | 1 | **0** | 1 | 1 | 1 | 1 | 0 | 0 | 0 | 0 | 0 |
| 20 | 9 | **6** | 3 | 3 | 3 | 3 | 6 | 6 | 9 | 3 | 3 |
| 21 | 0 | **1** | 1 | 1 | 1 | 1 | 1 | 1 | 1 | 1 | 1 |
| 22 | 4 | **3** | 2 | 3 | 4 | 4 | 4 | 3 | 4 | 4 | 4 |
| 24 | 1 | **1** | 1 | 1 | 1 | 1 | 1 | 1 | 1 | 1 | 1 |
| 25 | 0 | **0** | 0 | 1 | 1 | 4 | 5 | 5 | 3 | 4 | 2 |
| 31 | 3 | **3** | 1 | 3 | 5 | 3 | 11 | 6 | 6 | 5 | 6 |
| 32 | 4 | **2** | 1 | 4 | 5 | 8 | 8 | 11 | 9 | 7 | 5 |
| 33 | 1 | **1** | 1 | 1 | 1 | 1 | 1 | 1 | 1 | 1 | 1 |
| 34 | 0 | **1** | 0 | 2 | 2 | 2 | 2 | 3 | 3 | 3 | 7 |
| 35 | 2 | **0** | 1 | 1 | 1 | 1 | 1 | 1 | 1 | 1 | 1 |
| 39 | 11 | **8** | 3 | 3 | 3 | 3 | 3 | 3 | 3 | 3 | 3 |
| 41 | 1 | **1** | 1 | 1 | 1 | 1 | 1 | 1 | 1 | 1 | 1 |
| 47 | 1 | **1** | 0 | 0 | 0 | 0 | 0 | 0 | 0 | 0 | 0 |
| 48 | 3 | **3** | 2 | 1 | 1 | 1 | 1 | 1 | 1 | 1 | 1 |
| 49 | 9 | **5** | 1 | 0 | 0 | 0 | 0 | 0 | 0 | 0 | 0 |
| 50 | 1 | **1** | 1 | 1 | 1 | 1 | 1 | 1 | 1 | 1 | 1 |
| 55 | 0 | **0** | 0 | 1 | 1 | 1 | 0 | 0 | 0 | 0 | 1 |
| 57 | 2 | **1** | 2 | 2 | 2 | 2 | 2 | 2 | 2 | 2 | 2 |
| 58 | 1 | **1** | 1 | 1 | 1 | 1 | 1 | 1 | 1 | 1 | 1 |
| 59 | 0 | **1** | 1 | 1 | 1 | 1 | 1 | 1 | 1 | 1 | 1 |
| 61 | 0 | **0** | 0 | 0 | 0 | 3 | 0 | 0 | 0 | 0 | 0 |
| 62 | 2 | **5** | 0 | 3 | 3 | 2 | 3 | 3 | 3 | 3 | 3 |
| 64 | 1 | **2** | 0 | 0 | 0 | 0 | 0 | 0 | 0 | 0 | 0 |
| 66 | 1 | **5** | 1 | 1 | 1 | 1 | 1 | 1 | 1 | 1 | 1 |
| 69 | 0 | **0** | 3 | 2 | 3 | 4 | 4 | 6 | 2 | 3 | 2 |
| 71 | 3 | **3** | 0 | 1 | 0 | 4 | 5 | 4 | 3 | 2 | 2 |
| 76 | 0 | **1** | 1 | 1 | 1 | 1 | 2 | 1 | 1 | 2 | 1 |
| 77 | 2 | **3** | 0 | 0 | 0 | 0 | 0 | 0 | 0 | 0 | 0 |
| 90 | 0 | **0** | 1 | 3 | 3 | 5 | 4 | 3 | 6 | 2 | 4 |
| 91 | 0 | **0** | 0 | 0 | 0 | 0 | 0 | 0 | 0 | 0 | 0 |

Note: *R. miehei* CAU432 appears in bold. Enzymes abbreviated based on CAZyme classification. GTs, glycosyltransferases. Fungal species are abbreviated as: R. org, *Rhizopus oryzae* RA 99-880; *R. mie,* *Rhizomucor miehei* CAU432; P. chr, *Phanerochaete chrysosporium*; N. cra, *Neurospora crassa* OR74A; P. ans, *Podospora anserine* S mat+; M. gri, *Magnaporthe grisea* 70-15; A. org, *Aspergillus oryzae* RIB40; A. nig, *Aspergillus niger* CBS 513.88**;** P. chr, *Penicillium chrysogenum* Wisconsin 54-1255; A. nid, *Aspergillus nidulans* FGSC A4; L. mac, *Leptosphaeria maculans* v23.1.3.

**Table S6 Comparison of the number of PLs of *R. miehei* CAU432 with those of other fungi**

| PL | R. org | R. mie | P. chr | N. cra | P. ans | M. gri | A. org | A. nig | P. chr | A. nid | L. mac |
| --- | --- | --- | --- | --- | --- | --- | --- | --- | --- | --- | --- |
| 1 | 0 | **0** | 0 | 1 | 4 | 2 | 12 | 6 | 5 | 8 | 9 |
| 3 | 0 | **0** | 0 | 1 | 2 | 1 | 3 | 0 | 1 | 5 | 6 |
| 4 | 0 | **0** | 0 | 1 | 1 | 1 | 4 | 2 | 3 | 4 | 4 |
| 7 | 0 | **0** | 0 | 0 | 0 | 0 | 1 | 0 | 0 | 0 | 0 |
| 8 | 1 | **0** | 1 | 0 | 0 | 0 | 0 | 0 | 0 | 0 | 0 |
| 9 | 0 | **0** | 0 | 0 | 0 | 0 | 1 | 0 | 0 | 1 | 0 |
| 11 | 0 | **0** | 0 | 0 | 0 | 0 | 0 | 0 | 0 | 1 | 0 |
| 14 | 5 | **2** | 3 | 0 | 0 | 0 | 0 | 0 | 0 | 0 | 0 |

Note: *R. miehei* CAU432 appears in bold. Enzymes abbreviated based on CAZyme classification. PLs, Polysaccharide lyases. Fungal species are abbreviated as: R. org, *Rhizopus oryzae* RA 99-880; *R. mie,* *Rhizomucor miehei* CAU432; P. chr, *Phanerochaete chrysosporium*; N. cra, *Neurospora crassa* OR74A; P. ans, *Podospora anserine* S mat+; M. gri, *Magnaporthe grisea* 70-15; A. org, *Aspergillus oryzae* RIB40; A. nig, *Aspergillus niger* CBS 513.88**;** P. chr, *Penicillium chrysogenum* Wisconsin 54-1255; A. nid, *Aspergillus nidulans* FGSC A4; L. mac, *Leptosphaeria maculans* v23.1.3.

**Table S7 Comparison of the number of CEs of *R. miehei* CAU432 with those of other fungi**

| CE | R. org | **R. mie** | P. chr | N. cra | P. ans | M. gri | A. org | A. nig | P. chr | A. nid | L. mac |
| --- | --- | --- | --- | --- | --- | --- | --- | --- | --- | --- | --- |
| 1 | 0 | **1** | 5 | 7 | 14 | 10 | 5 | 3 | 2 | 3 | 4 |
| 2 | 0 | **0** | 0 | 0 | 0 | 1 | 0 | 0 | 0 | 0 | 1 |
| 3 | 0 | **0** | 0 | 3 | 8 | 6 | 3 | 1 | 4 | 6 | 2 |
| 4 | 32 | **16** | 4 | 4 | 5 | 8 | 4 | 6 | 5 | 8 | 7 |
| 5 | 0 | **0** | 0 | 3 | 7 | 15 | 5 | 5 | 4 | 4 | 8 |
| 8 | 6 | **2** | 2 | 1 | 1 | 1 | 5 | 3 | 2 | 3 | 3 |
| 9 | 1 | **1** | 1 | 1 | 1 | 1 | 1 | 1 | 1 | 1 | 2 |
| 10 | 0 | **0** | 0 | 0 | 0 | 0 | 0 | 0 | 0 | 0 | 0 |
| 12 | 0 | **0** | 0 | 1 | 1 | 2 | 4 | 2 | 2 | 2 | 3 |
| 15 | 0 | **0** | 3 | 1 | 3 | 1 | 0 | 0 | 1 | 0 | 2 |
| 16 | 0 | **0** | 1 | 1 | 1 | 1 | 3 | 2 | 1 | 3 | 2 |

Note: *R. miehei* CAU432 appears in bold. Enzymes abbreviated based on CAZyme classification. CEs, carbohydrate esterases. Fungal species are abbreviated as: R. org, *Rhizopus oryzae* RA 99-880; *R. mie,* *Rhizomucor miehei* CAU432; P. chr, *Phanerochaete chrysosporium*; N. cra, *Neurospora crassa* OR74A; P. ans, *Podospora anserine* S mat+; M. gri, *Magnaporthe grisea* 70-15; A. org, *Aspergillus oryzae* RIB40; A. nig, *Aspergillus niger* CBS 513.88**;** P. chr, *Penicillium chrysogenum* Wisconsin 54-1255; A. nid, *Aspergillus nidulans* FGSC A4; L. mac, *Leptosphaeria maculans* v23.1.3.

**Table S8 Comparison of the number of CBMs of *R. miehei* CAU432 with those of other fungi**

| CBM | R. org | R. mie | P. chr | N. cra | P. ans | M. gri | A. org | A. nig | P. chr | A. nid | L. mac |
| --- | --- | --- | --- | --- | --- | --- | --- | --- | --- | --- | --- |
| 1 | 3 | **2** | 31 | 19 | 30 | 22 | 3 | 8 | 6 | 7 | 5 |
| 5 | 2 | **3** | 3 | 0 | 0 | 0 | 0 | 0 | 0 | 0 | 0 |
| 6 | 0 | **0** | 0 | 0 | 1 | 2 | 0 | 0 | 0 | 0 | 0 |
| 12 | 2 | **1** | 0 | 0 | 0 | 0 | 0 | 0 | 0 | 0 | 0 |
| 13 | 2 | **6** | 5 | 2 | 0 | 0 | 2 | 1 | 0 | 0 | 0 |
| 14 | 0 | **0** | 0 | 0 | 0 | 0 | 1 | 1 | 1 | 1 | 0 |
| 18 | 0 | **0** | 1 | 3 | 32 | 30 | 5 | 13 | 12 | 17 | 29 |
| 19 | 3 | **1** | 0 | 0 | 0 | 0 | 1 | 0 | 0 | 0 | 0 |
| 20 | 0 | **0** | 2 | 2 | 3 | 3 | 1 | 1 | 3 | 4 | 3 |
| 21 | 8 | **2** | 2 | 1 | 1 | 1 | 1 | 1 | 1 | 1 | 1 |
| 24 | 0 | **0** | 0 | 8 | 2 | 0 | 6 | 6 | 8 | 2 | 0 |
| 32 | 0 | **0** | 0 | 1 | 0 | 0 | 2 | 0 | 2 | 0 | 1 |
| 35 | 0 | **0** | 1 | 0 | 2 | 3 | 1 | 2 | 1 | 2 | 3 |
| 38 | 0 | **0** | 0 | 1 | 0 | 0 | 0 | 0 | 0 | 0 | 0 |
| 42 | 0 | **0** | 0 | 0 | 1 | 1 | 1 | 1 | 1 | 1 | 0 |
| 43 | 2 | **1** | 1 | 1 | 1 | 1 | 5 | 4 | 3 | 3 | 2 |
| 48 | 2 | **2** | 1 | 1 | 3 | 1 | 2 | 3 | 2 | 1 | 2 |
| 50 | 3 | **2** | 1 | 2 | 19 | 0 | 3 | 2 | 9 | 2 | 5 |
| 52 | 0 | **0** | 0 | 1 | 2 | 1 | 0 | 0 | 0 | 0 | 0 |

Note: *R. miehei* CAU432 appears in bold. Enzymes abbreviated based on CAZyme classification. CBMs, carbohydrate-binding modules. Fungal species are abbreviated as: R. org, *Rhizopus oryzae* RA 99-880; *R. mie,* *Rhizomucor miehei* CAU432; P. chr, *Phanerochaete chrysosporium*; N. cra, *Neurospora crassa* OR74A; P. ans, *Podospora anserine* S mat+; M. gri, *Magnaporthe grisea* 70-15; A. org, *Aspergillus oryzae* RIB40; A. nig, *Aspergillus niger* CBS 513.88**;** P. chr, *Penicillium chrysogenum* Wisconsin 54-1255; A. nid, *Aspergillus nidulans* FGSC A4; L. mac, *Leptosphaeria maculans* v23.1.3.

**Table S9 Numbers of peptidase genes in *R. miehei* and *Aspergillus oryzae***

|  | EC number | Number of genes | |
| --- | --- | --- | --- |
|  |  | *Rhizomucor miehei* | *Aspergillus oryzae* |
| Exopeptidase |  |  |  |
| Aminopeptidase | 3.4.11.- | 18 | 19 |
| Dipeptidase | 3.4.13.- | 6 | 3 |
| Dipeptidyl of tripeptidyl peptidase | 3.4.14.- | 4 | 9 |
| Serine-type carboxypeptidase | 3.4.16.- | 5 | 12 |
| Metallocarboxypeptidase | 3.4.17.- | 8 | 12 |
| Omega peptidase | 3.4.19.- | 2 |  |
| Unknown |  |  | 14 |
| Total |  | 43 | 69 |
| Endopeptidase |  |  |  |
| Serine endopeptidase | 3.4.21.- | 36 | 11 |
| Cysteine endopeptidase | 3.4.22.- | 10 | 12 |
| Aspartic endopeptidase | 3.4.23.- | 18 | 14 |
| Metalloendopeptidase | 3.4.24.- | 33 | 18 |
| Threonine endopeptidase | 3.4.25.- | 14 | - |
| Unknown | 3.4.99.- | 1 | 10 |
| Total |  | 112 | 65 |
| Exopeptidase + Endopeptidase |  | 155 | 134 |
