# Supplementary material for: Genome sequence and transcriptome analyses of the thermophilic zygomycete fungus Rhizomucor miehei
Source: BMC Genomics. 2014 Apr 21;15:294. doi: 10.1186/1471-2164-15-294 (PMC4023604; doi:10.1186/1471-2164-15-294)
Supplement: Additional file 2: Figure S1 — Location of various enzymes in R. miehei CAU432. Figure S2. RNA-seq analysis of R. miehei transcriptome from mycelia growing at 30°C and 50°C. Figure S3. Gene expression in R. miehei CAU432 mycelia grown at 30°C (labeled as T30) and 50°C (labeled as T50). Figure S4. Gene expression changes in mycelia of R. miehei CAU432 growth at 50°C comparing with that of growth at 30°C. Figure S5. Molecular functions of Gene Ontology for differently expressed genes with at least two-fold changes between two growth temperatures in R. miehei CAU432. [file 1471-2164-15-294-S2.doc]

**Additional files**

**Figure caption**

**Figure S1** **Location of various enzymes in *R. miehei* CAU432.** Membrane proteins were distributed in both of the secretory and non-secretory proteins.

**Figure** S**2 RNA-seq analysis of *R. miehei* transcriptome from mycelia growing at 30 °C and 50 °C.** (a) Distribution of reads mapped to the predicted genes expressed as natural logarithm Log_2_ (read counts for each gene) of the mycelia growing at 50 °C vs 30 °C. (b) CDS: CoDing Sequence for amino acids in protein; Reads uniquely mapped to exon and intron ranges of predicted genome genes, splice junctions (SJ), antisense strands and other regions were indicated. (c) Coverage distribution by RNA-seq reads from transcriptome of 30 °C and 50 °C mapped to the predicted genes of *R. miehei* genome, X axis is the coverage range and Y axis is the counts of the nucleotide whose coverage fall into the coverage range. (d) MA-plot of differentially expressed genes (red points) between mycelia growth at 30 °C and at 50 °C was plotted with DEGseq. The red lines show the “theoretical” four-fold local standard deviation of Maccording to the random sampling model. M = log_2_C_1_− log_2_C_2_, and A = (log_2_C_1_ + log_2_C_2_)/2; C_1_: of 50 °C; C_2_: of 30 °C.

**Figure S3** **Gene expression in *R. miehei* CAU432 mycelia grown at 30 °C (labeled as T30) and 50 °C (labeled as T50).** **(**a**)** Venn diagrams of the gene numbers of unique and shared transcripts in the nonnormalized mycelia grown at 30 °C and 50 °C. **(**b**)** Comparison of up- and down-regulated gene numbers in 8 different groups of genes.

**Figure S4** **Gene expression changes in mycelia of *R. miehei* CAU432 growth at 50 °C comparing with that of growth at 30 °C.** Heat map of expression of the *R. miehei* at the two different temperatures. The bar at the right side of the grouped gene panels represents relative expression values. Only annotated genes with expression changes over 1 fold are clustered and displayed in green-red color gradient.

**Figure S5** **Molecular functions of Gene Ontology for differently expressed genes with at least two-fold changes between two growth temperatures in *R. miehei* CAU432.** Brown bars denote the number of down-regulated genes and blue bars for the up-regulated genes at 50 °C, compared to 30 °C.


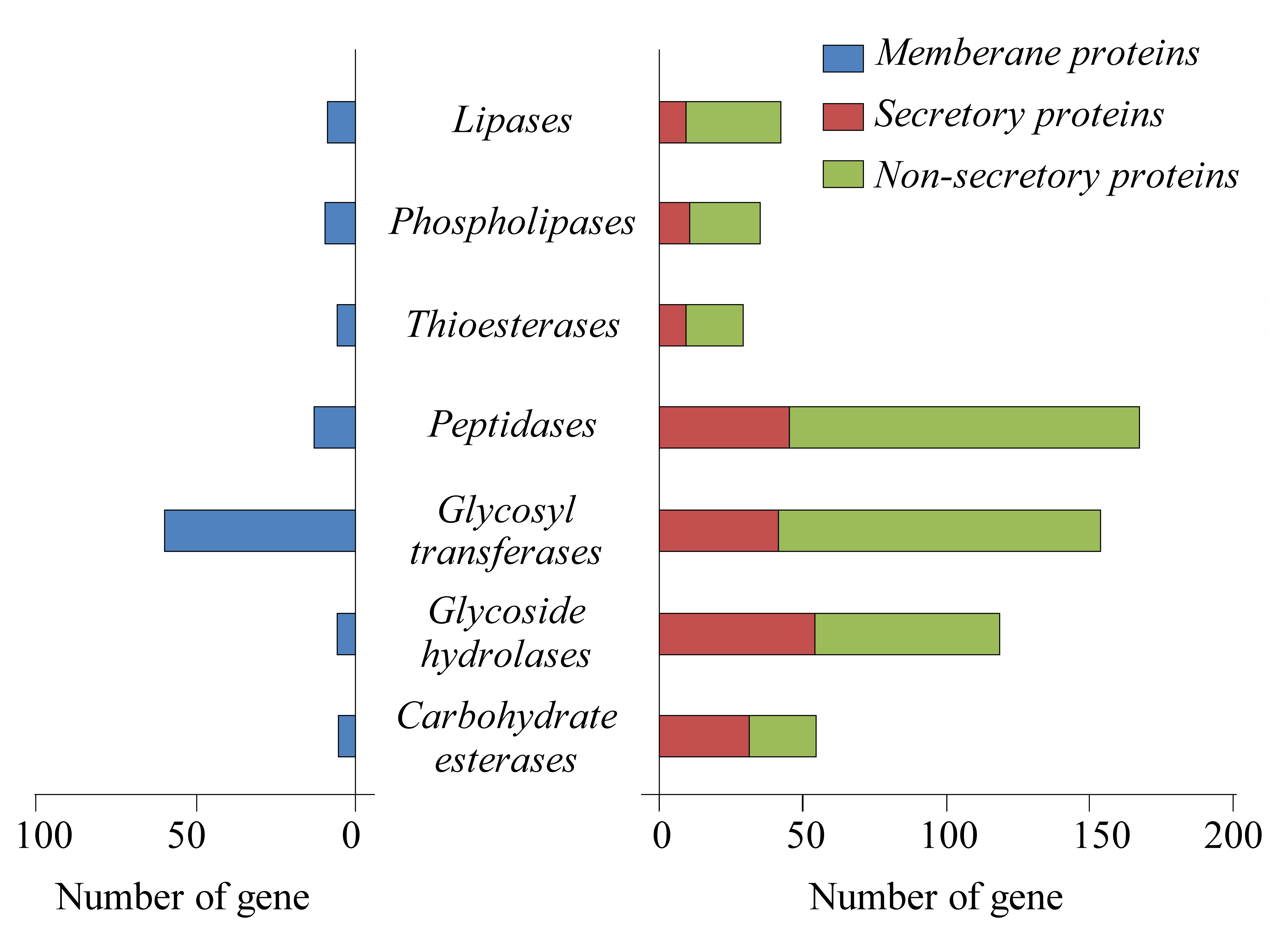
**Figure S1**


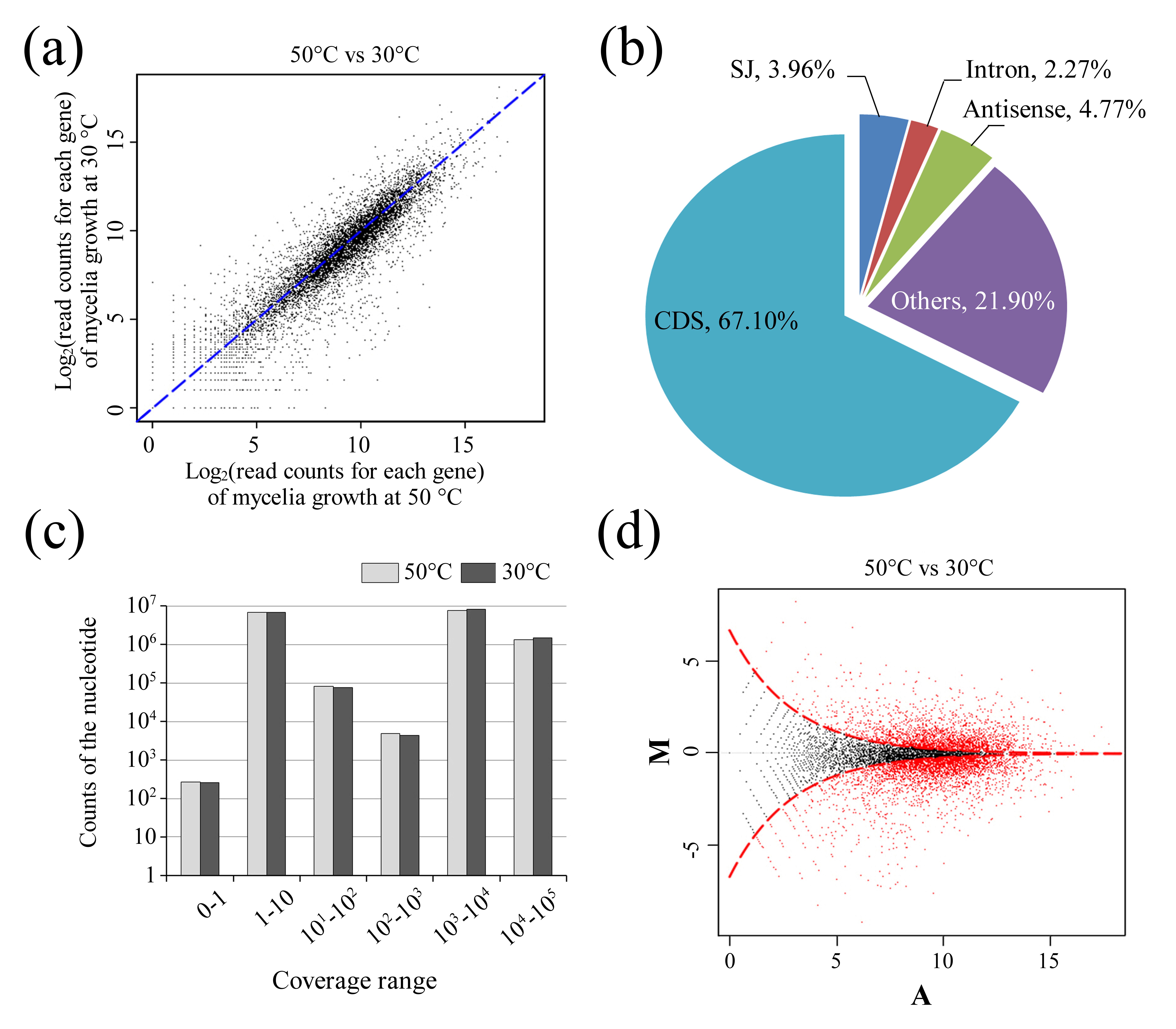
**Figure S2**


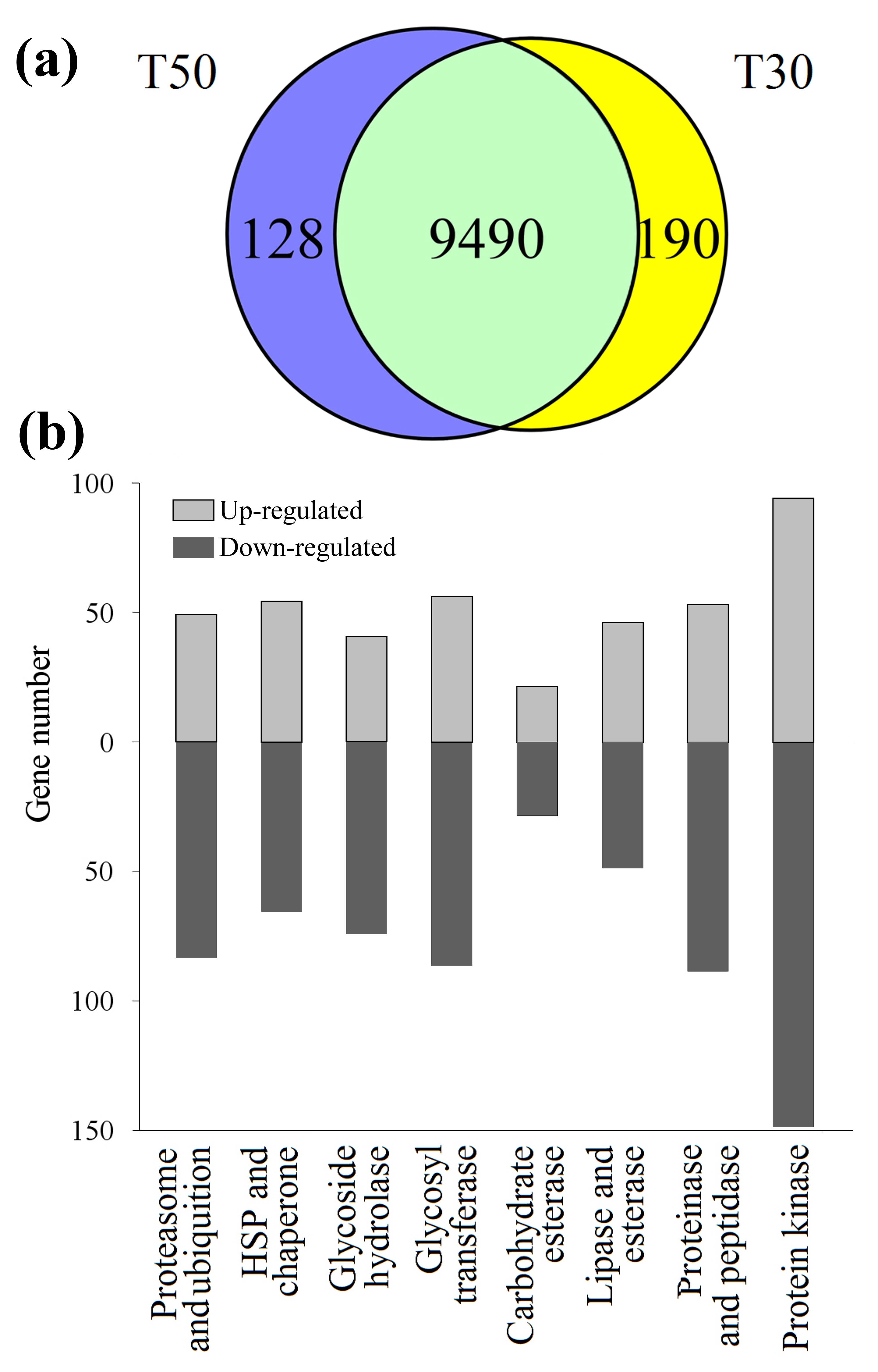
 **Figure S3**

**
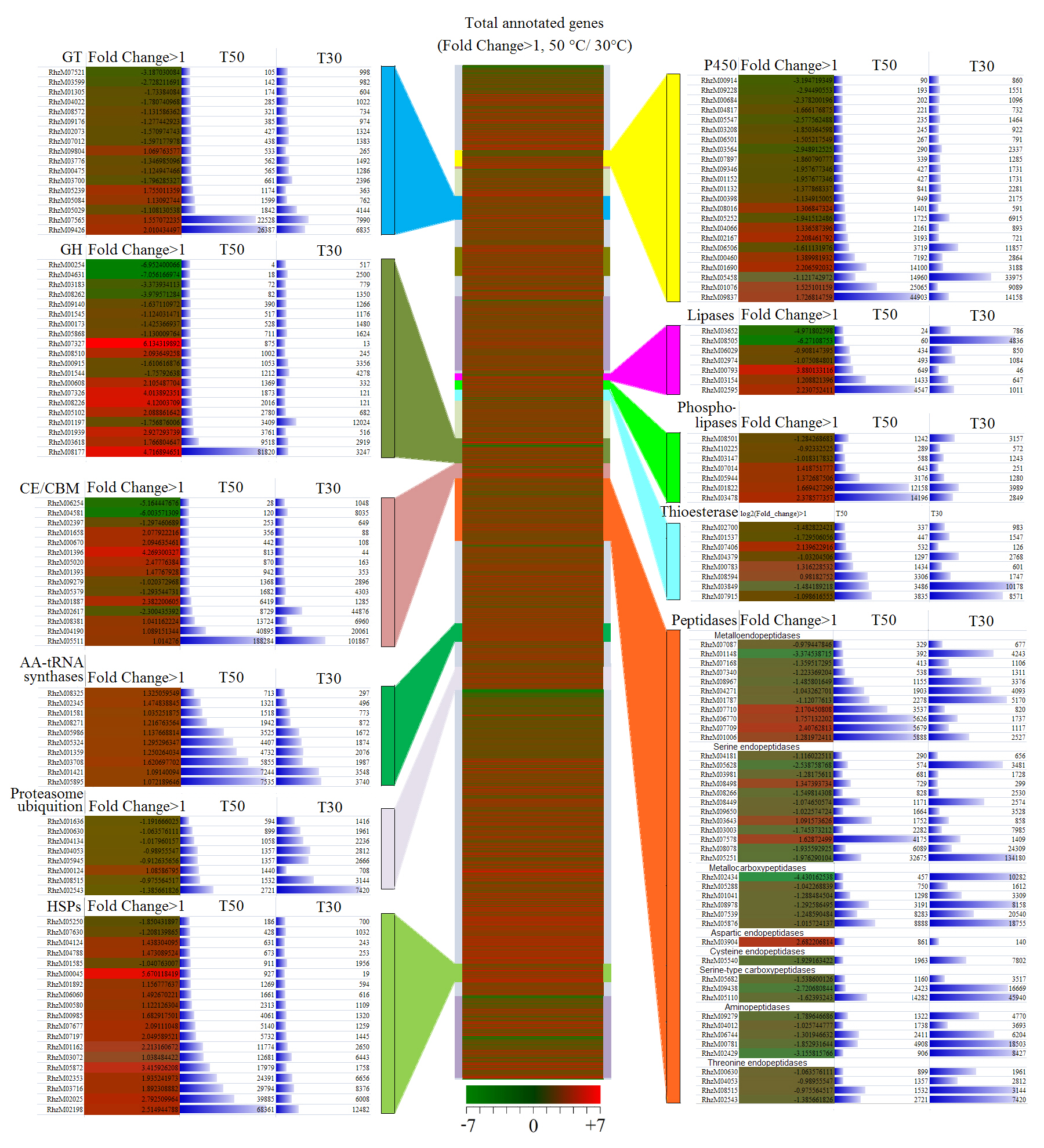
**

**Figure S4**

**
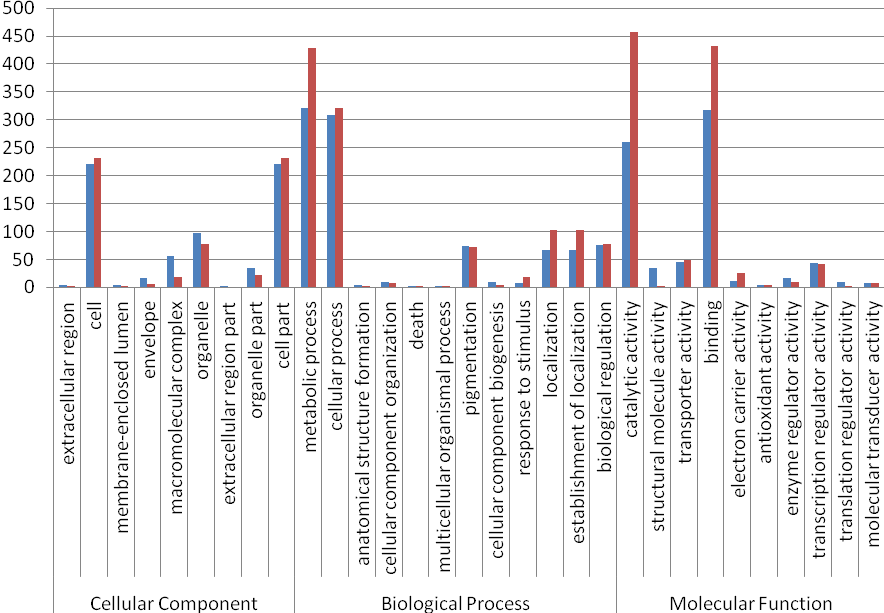
**

Number of genes

**Figure S5**
